# *G6PD* and machine learning algorithms as prognostic and diagnostic indicators of liver hepatocellular carcinoma

**DOI:** 10.1186/s12885-024-11887-6

**Published:** 2024-01-31

**Authors:** Fei Li, Boshen Wang, Hao Li, Lu Kong, Baoli Zhu

**Affiliations:** 1 Key Laboratory of Environmental Medicine Engineering of Ministry of Education, School of Public Health, Southeast University, 87 Dingjiaqiao, Nanjing, 210009 Jiangsu China; 2 Institute of Occupational Disease Prevention, Jiangsu Provincial Center for Disease Prevention and Control, Nanjing, Jiangsu 210009 China; 3 Jiangsu Preventive Medical Association, Nanjing, 210000 Jiangsu China; 4 Center for Global Health, Nanjing Medical University, Nanjing, 211112 China; 5 Jiangsu Province Engineering Research Center of Public Health Emergency, Nanjing, 210000 Jiangsu China

**Keywords:** *G6PD*, Liver hepatocellular carcinoma, Prognostic, Machine learning, Immunology, Drug Sensitivity, Cell proliferation, Cell migration

## Abstract

**Background:**

Liver Hepatocellular carcinoma (LIHC) exhibits a high incidence of liver cancer with escalating mortality rates over time. Despite this, the underlying pathogenic mechanism of LIHC remains poorly understood.

**Materials & methods:**

To address this gap, we conducted a comprehensive investigation into the role of *G6PD* in LIHC using a combination of bioinformatics analysis with database data and rigorous cell experiments. LIHC samples were obtained from TCGA, ICGC and GEO databases, and the differences in *G6PD* expression in different tissues were investigated by differential expression analysis, followed by the establishment of Nomogram to determine the percentage of *G6PD* in causing LIHC by examining the relationship between *G6PD* and clinical features, and the subsequent validation of the effect of *G6PD* on the activity, migration, and invasive ability of hepatocellular carcinoma cells by using the low expression of LI-7 and SNU-449. Additionally, we employed machine learning to validate and compare the predictive capacity of four algorithms for LIHC patient prognosis.

**Results:**

Our findings revealed significantly elevated *G6PD* expression levels in liver cancer tissues as compared to normal tissues. Meanwhile, Nomogram and Adaboost, Catboost, and Gbdt Regression analyses showed that *G6PD* accounted for 46%, 31%, and 49% of the multiple factors leading to LIHC. Furthermore, we observed that *G6PD* knockdown in hepatocellular carcinoma cells led to reduced proliferation, migration, and invasion abilities. Remarkably, the Decision Tree C5.0 decision tree algorithm demonstrated superior discriminatory performance among the machine learning methods assessed.

**Conclusion:**

The potential diagnostic utility of *G6PD* and Decision Tree C5.0 for LIHC opens up a novel avenue for early detection and improved treatment strategies for hepatocellular carcinoma.

**Supplementary Information:**

The online version contains supplementary material available at 10.1186/s12885-024-11887-6.

## Introduction

Liver Hepatocellular carcinoma (LIHC) has a very high incidence of liver cancer. Meanwhile, liver cancer ranks third among cancer deaths [1]. In 2012, there were more than 700,000 confirmed cases of Hepatocellular carcinoma in China, and the number of death cases also exceeded 700,000. The incidence and mortality of LIHC continue to rise worldwide, including in China [1–3]. Because the clinical symptoms of LIHC are not easily detected, 80% of patients are not well treated. Better treatment options are urgently needed for patients with LIHC [4].

*G6PD* is a very important biomarker in the pentose phosphate pathway (PPP), which is a precursor for nicotinamide adenine dinucleotide phosphate (NADPH) production in tumor cells [5]. Research has shown that high expression of *G6PD* is strongly correlated to the poor clinical prognosis of bladder cancer, lung cancer, and breast cancer [6–8]. Animal studies have shown a relationship between *G6PD* and precancerous lesions in rat liver. The high expression of *G6PD* increased the incidence of precancerous lesions, and the number and volume of LIHC cells were also higher than those in the low expression group of *G6PD* [9]. Nevertheless, the specific process of *G6PD* participating in LIHC needs to be further studied.

In recent years, clinicians have applied machine learning to cancer diagnosis and prognosis prediction, which has significantly improved the survival rate of cancer patients [10, 11]. Through machine learning, clinicians can use big data analysis to analyze a large amount of clinical data, accurately predict the prognosis of patients, and facilitate the finding of feasible treatment methods and symptomatic treatment [12]. Therefore, a large number of Machine learning algorithms have been developed, such as Bayesian Classifier, Neural network algorithm, Support vectors machine and Decision Tree C5.0 [13–15]. However, the application performance of machine learning in LIHC has not been validated.

In this study, data analysis investigated the possible mechanism between *G6PD* and LIHC. We concluded that the expression level of *G6PD* could be regarded as a prognostic and diagnostic criterion for LIHC patients. Gene correlation studies, immunoassays, and drug sensitivity analysis have provided new ideas for treating LIHC. Finally, we compared the ability of four machine learning algorithms to distinguish between LIHC and para-cancerous tissues.

## Materials and methods

### Datasets acquisition

**Fig. 1:**
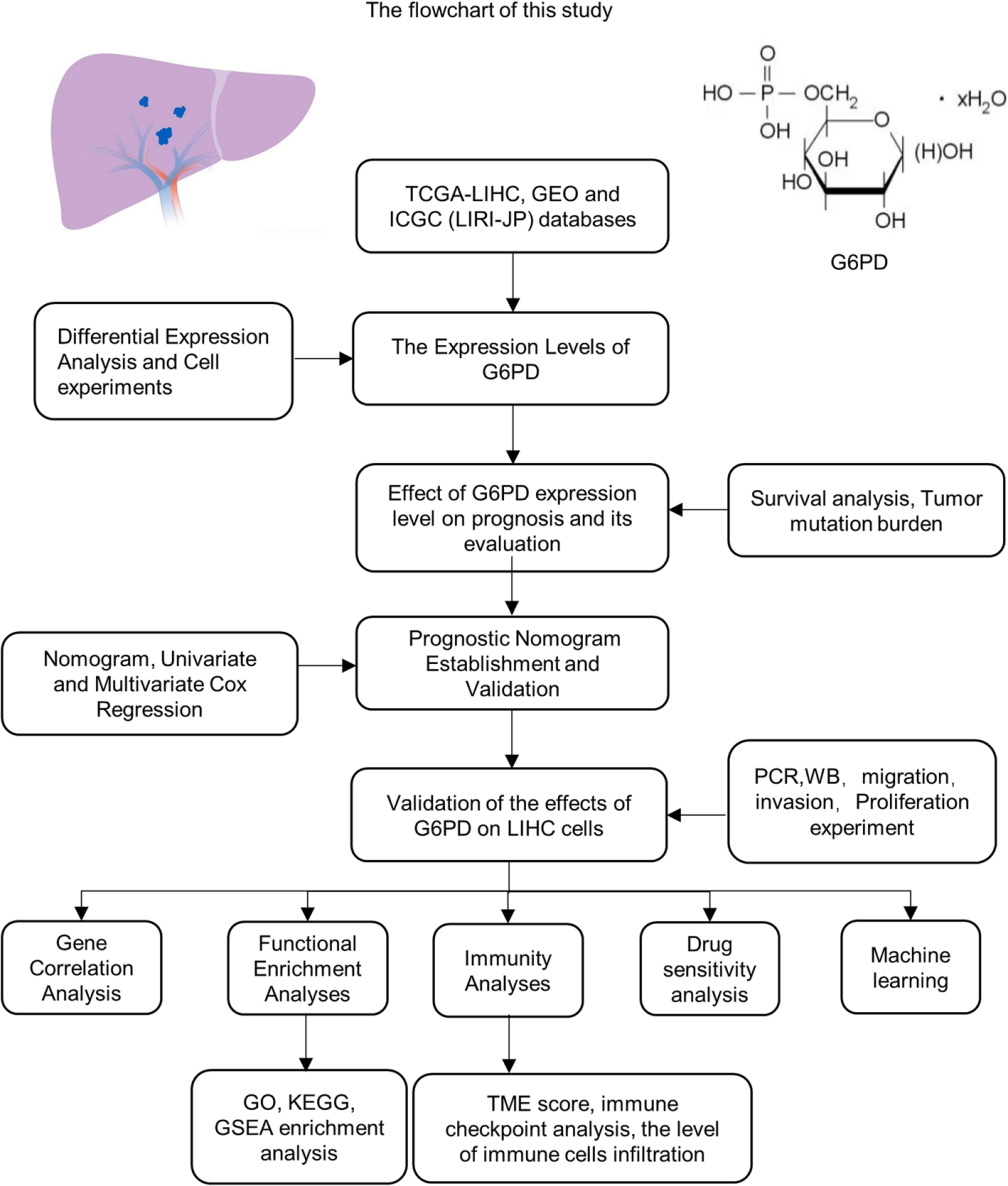
The flowchart of this study

We obtained related clinical information and the RNA sequencing data of LIHC patients from the Cancer Genome Atlas (TCGA) database (https://portal.gdc.cancer.gov/projects/TCGA-LIHC, dbGaP Study Accession:phs000178, 26/7/2022, human), the International Cancer Genome Consortium (ICGC-LIRI-JP cohort, https://dcc.icgc.org/projects/LIRI-JP, 28/8/2022, human) database and the Gene Expression Omnibus (GEO) database (http://www.ncbi.nlm.nih.gov/geo/,28/8/2022, human). The TCGA database was elected as a training cohort and the databases from ICGC (LIRI-JP) and GEO (GSE14520, GSE20140, GSE62232, GSE84005) as validation cohorts. It was verified that the *G6PD* gene expression level data obeyed a normal distribution, subsequently, we matched the clinical data and gene expression of each patient in each database, and then classified those with *G6PD* expression above the average level as high expression group, and those with *G6PD* expression below the average level as low expression group. Figure 1 is the flow chart of this experimental study.

### Differential expression analysis and verification of protein expressions of *G6PD*

The Bioconductor package edgeR was employed to identify the differential expression levels of *G6PD* in different cancer and normal tissues. Immunohistochemistry (IHC) is an approach to detecting the distribution and expression of relative proteins based on the specific binding of antigens to antibodies. We captured the prognosis-related protein expression profiles of the *G6PD* gene from the Protein Atlas (HPA, https://www.proteinatlas.org/) database for hepatocellular carcinoma tissues and normal tissues.

### Correlation analysis between *G6PD* expression level and clinical characteristics

Cox regression analyses were fulfilled to value the connection between traditional clinical characteristics (including Age, Gender, Grade, TNM stage, and Tumor stage I-IV) and *G6PD* expression level, which provided the basis for the establishment of the Nomogram.

### Nomogram construction and verification

A nomogram is a method to predict the clinical outcome of LIHC patients. R software was used to construct a Nomogram including Age, Gender, Grade, Stage, and *G6PD*. In addition, the receiver operating characteristic (ROC) curve and the calibration curves were drawn to score the prediction accuracy of the Nomogram.

### Assessment of the importance of G6PD in LIHC

To further assess the value of *G6PD* as a clinical prognostic indicator of hepatocellular carcinoma, three machine learning models, Adaboost regression, Catboost Regression, and Gbdt Regression, were utilized to appraise the importance of *G6PD* in the prognosis of patients with LIHC.

### Gene correlation analysis

The R software was utilized to probe the correlation between *CDC20, CEP55, TRIP13, MYBL2* and *G6PD*. Gene correlation analysis was used to explore the valuable genes that may be related to *G6PD*, this could point to the possibility that these genes may have biologically similar functions, participate in the same pathways, or be similarly regulated.

### GO, KEGG, and GSEA analysis

In order to understand the cellular component, molecular function, and biological processes of *G6PD* in humans, the Gene Ontology (GO) was performed by the R software. Meanwhile, the Kyoto Encyclopedia of Genes and Genomes (KEGG) pathway analysis and Gene-set enrichment analysis (GSEA) were implemented to comprehend the related signaling pathways of *G6PD*.

### Drug sensitivity assessment

Drug sensitivity data for LIHC patients is available in the Cancer Drug Sensitivity Genomics Database (https://www.cancerrxgene.org/). the drug response was presented by the half-maximal inhibitory concentration (IC50) which used the R software. The results have appeared in box plots.

### Calculation of tumor mutational burden

Tumor mutation load (TMB) is an index used to reflect the ability and degree of the tumor to produce new antigens, which can indirectly predict the effect of immunotherapy on all kinds of tumors. A high TMB index indicates a better clinical immunotherapy effect. The TMB values were calculated using Perl scripts, and the above results were displayed in a scatter diagram.

### Immunological analysis

To understand the relationship between Tumor Microenvironment (TME) and *G6PD*, we plotted the violin of Stromal score, the immune scores, and the estimate scores in two groups of LIHC patients with high and low *G6PD* expression. We used the CIBERSORT algorithm to calculate differences in 22 tumor-infiltrating immune cells in LIHC patients with high and low *G6PD* expression levels. CTLA4 and PD-1 Immune checkpoint is commonly used in immunotherapy. Finally, we obtained the immunophenotype (IPS) of LIHC from the LIHC project of Cancer Immunoomics Atlas (TCIA, https://www.tcia.at/home) to predict the response to immunotherapy in the group with high and low *G6PD* expression.

### Survival analysis

Clinical data from TCGA database, GEO (GSE14520) database and ICGC database were analyzed for survival by Kaplan–Meier curve survival analysis using R software. Overall survival (OS) and progression-free survival (PFS) were analyzed for TCGA data, and OS was analyzed for GEO (GSE14520) database and ICGC database.

### Feasibility of machine learning algorithms in clinical prognosis of LIHC patients

In order to accurately predict a patient's prognosis, many machine learning algorithms have been developed in recent years. We selected four machine learning algorithms for comparison: Bayesian classifier, Neural network algorithm, Support vectors machine and Decision Tree C5.0.

### Cell culture and transfection

We purchased WRL68 liver normal cells from Shanghai Fuheng Biotechnology Co., LTD. (Fuheng, Shanghai, China), LI-7, SNU-398, SNU-449, SK-HEP-1 Liver cancer cells were acquired from Guangzhou Cellcook Biotechnology Co., LTD (Cellcook, Guangzhou, China). Dulbecco’s modified Eagle’s medium (DMEM; Gibco, Shanghai, China) was used to culture WRL68. RPMI 1640’s modified Eagle’s medium was used to culture SNU-398 (RPMI 1640 modified; Gibco, Shanghai, China). Meanwhile, we cultured LI-7, SNU-449, SK-HEP-1 cells using RPMI 1640 medium. siRNA-*G6PD* and non-targeting control siRNA (NC-siRNA) were purchased from Ribobio Biotechnology Co., LTD. (Ribobio, Guangzhou, China). The sequence of siRNA-G6PD was TCCTCTATGTGGAGAATGA. The stareffect II transfection reagent (GenStar, Beijing, China) was bound to siRNA-*G6PD* or NC-SIRRNA for 10 min and transfected into LI-7 or SNU-449 cells. The solution was changed after 6 h, and cells were collected after 48 h.

### Quantitative real-time PCR (qRT-PCR)

Total RNA from WRL68, LI-7, SNU-398, SNU-449, SK-HEP-1 cells was extracted using RNA-easy Isolation Reagent (Vazyme, Nanjing, China). For qRT-PCR, the following primers were used: human *G6PD*, 5′-AAGAACGTGAAGCTCCCTGA-3′ (Forward) and 5′-AATATAGGGGATGGGCTTGG-3′ (Reverse); human β-actin, 5′-GGAAATCGTGCGTGACAT-3′ (Forward) and 5′-GGTGATGACCTGGCCGTT-3′ (Reverse). Relative expression of *G6PD* was analyzed using the 2 − ΔΔCT method.

### Western blots

The RIPA buffer (GenStar, Beijing, China) was used to lyse the cells and obtain the protein, which was then measured by BCA and a protein sample was made. The proteins were transferred into the membrane by electrophoresis and membrane transfer steps. Then it was sealed with a sealing solution for 1h, incubated with primary antibody for 8h-12h, incubated with secondary antibody for 1h, and developed by chemiluminescence. Primary antibodies were: Recombinant Anti-Glucose 6 Phosphate Dehydrogenase antibody [EPR20668] (Abcam, ab210702), and Anti-beta Actin [mAbcam 8226] (Abcam, ab8226).

### Cell viability

LI-7 and SNU-449 were inoculated into 96-well plates with 2000 cells/well and incubated for 24 h to attach to the wall. 48 h after transfection with siRNA-*G6PD* or NC-siRNA, fresh medium and 10 μL cell counting Kit 8 (CCK-8, Beyotime, China) were added to each well, and after incubation at 37°C for 1 h, absorbance at 450 nm was obtained by spectrophotometer and cell viability was analyzed.

### Transwell invasion and migration assay

Cell chambers were placed in 24-well plates and invasion and migration experiments were performed with or without BD Matrigel TM (BD Bioscience, USA). Migration experiment: 0.3 mL serum-free medium containing 2 × 105 cells was added to the upper part of the cell chamber and 0.7ml medium containing 10% serum was added to the lower part of the cell chamber for 12 and 24 h. Invasion experiment: 0.3 mL serum-free medium containing 4 × 105 cells was added to the upper part of the cell chamber and 0.7ml medium containing 10% serum was added to the lower part of the cell chamber for 24 and 48 h. First, cells were fixed with 4% paraformaldehyde for 10 min, then stained with 0.5% crystal violet for 30 min, cleaned and wiped away the excess cells.

### Statistical analysis

All statistical data were analyzed using R software (4.0.5). Univariate and multivariate Cox regression analyses were applied to appraise the association between *G6PD* and different clinical features. Log-rank test and Kaplan–Meier analysis were used to assess the effect of *G6PD* expression level on the survival status of LIHC patients. ROC curve was applied to evaluate the performance of the Nomogram and the machine learning algorithm. the statistical significance was installed at *P* < 0.05.

## Results

### The expression level of *G6PD*

**Fig. 2:**
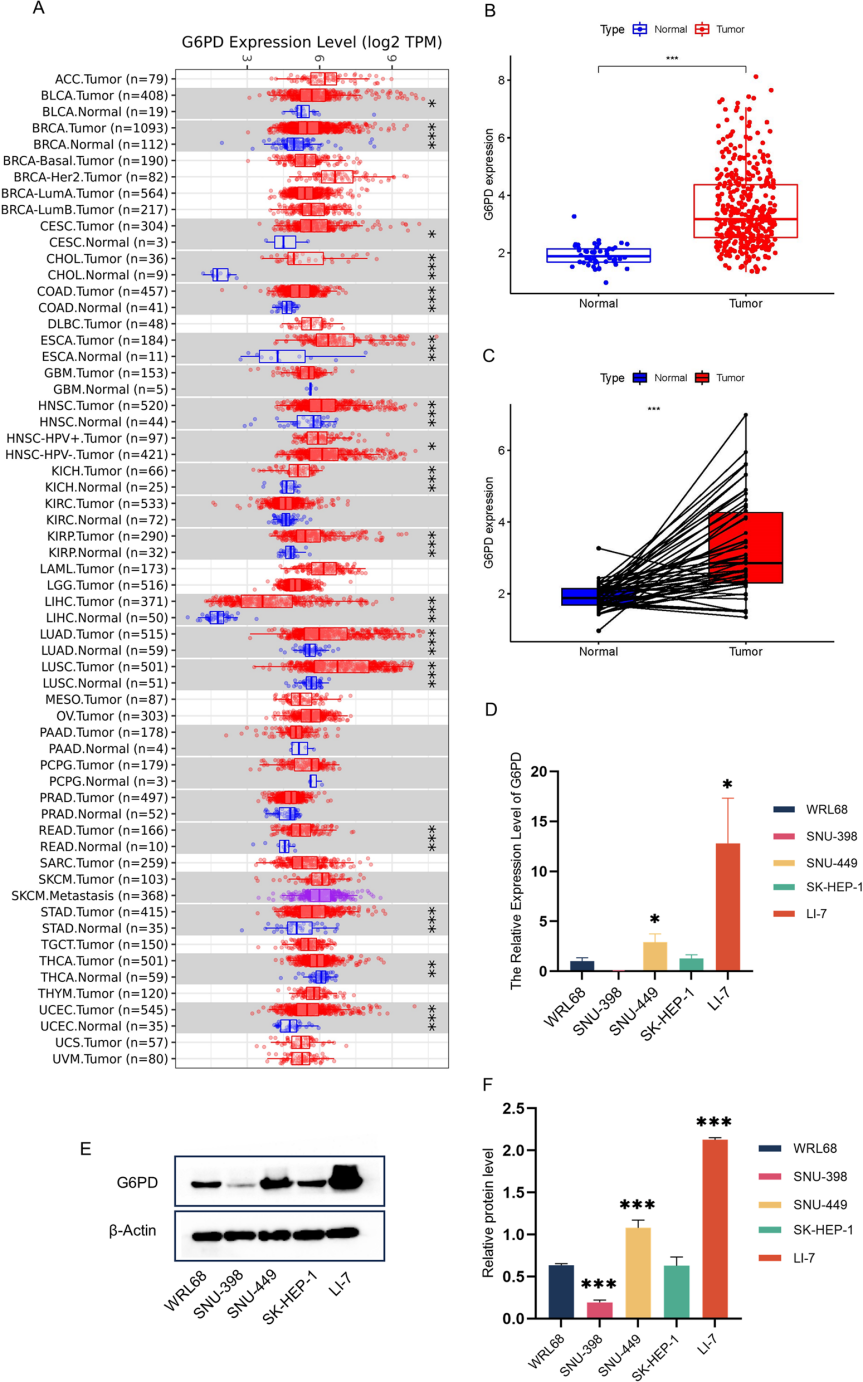
The Expression level of *G6PD*. **A** The different expression level of *G6PD* between normal and tumor tissues in TCGA. **B** The different expression level of *G6PD* between normal and hepatocellular carcinoma tissues in TCGA. **C** The different expression level of *G6PD* between normal and hepatocellular carcinoma tissues in the same patient in TCGA. **D**-**F** PCR and WB results of the expression level of *G6PD* in normal hepatocytes and hepatoma cells. *P < 0.05, **P < 0.01, ***P < 0.001

First of all, we analyzed the discrepancy of *G6PD* expression levels between different cancer tissues and normal tissues, and there were significant differences in *G6PD* in 17 tissues. LIHC was selected as the research direction of this paper (Fig. 2A). Then, we contrasted the expression levels of *G6PD* between normal Hepatocellular tissue and LIHC tissue. The expression level of *G6PD* in LIHC tissue was high, and LIHC tissues of the same person tended towards higher *G6PD* expression levels than normal tissues (Fig. 2B-C). the results of the ICGC and GSE databases were consistent with those of the TCGA database (Fig. S1A-D). the HPA database showed that *G6PD* was highly expressed in LIHC tissues (Fig. S1E-F). Subsequently, we analyzed the *G6PD* gene expression in WRL68, LI-7, SNU-398, SNU-449, SK-HEP-1 cells, and showed that compared with normal liver cells, the *G6PD* expression level of liver cancer cells was significantly increased, and the expression level of LI-7 and SNU-449 was the highest (Fig. 2D). the results of Western blots were the same as those of PCR (Fig. 2E-F).

### Effect of *G6PD* expression levels on prognosis

**Fig. 3:**
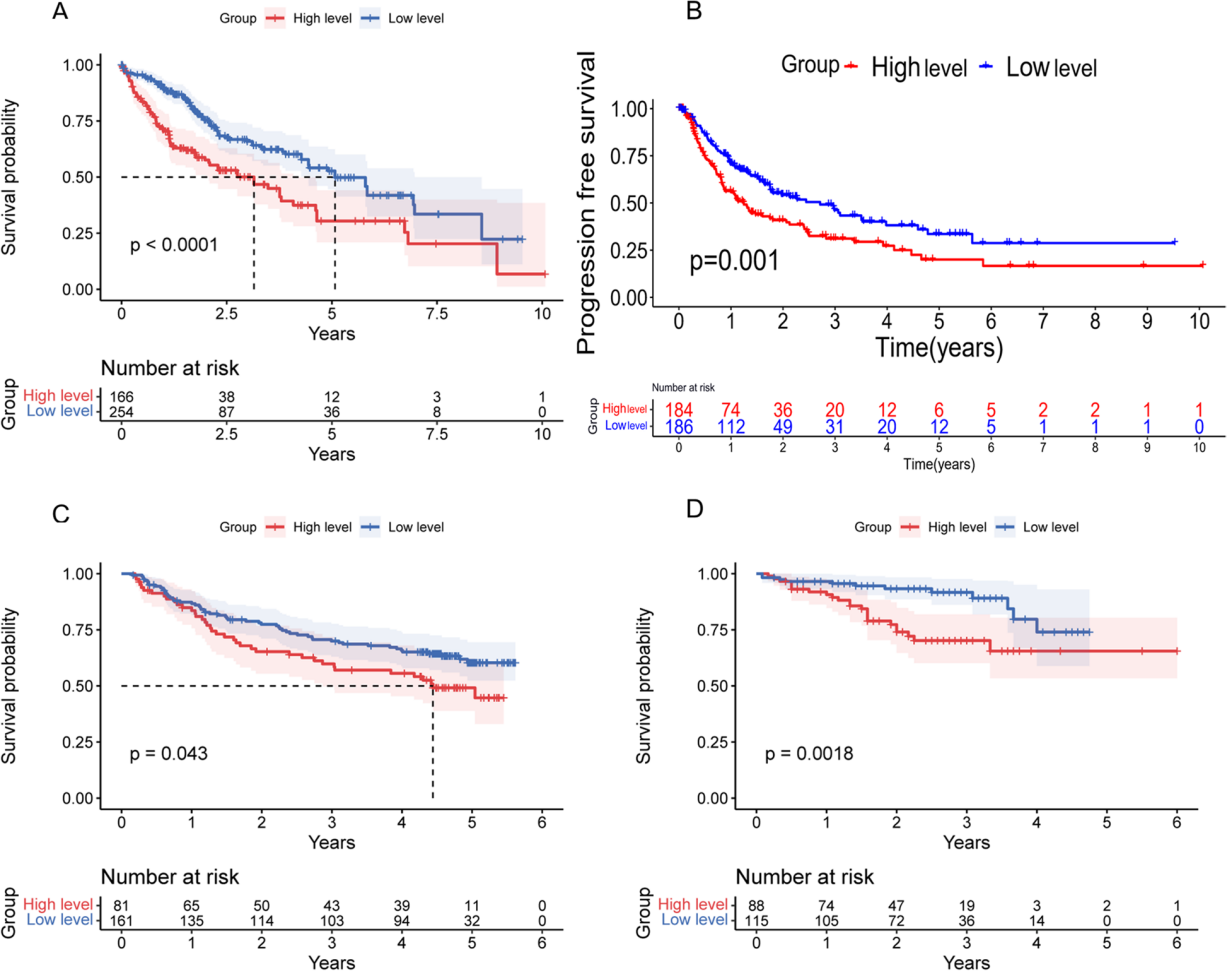
Effect of G6PD Expression Level on Prognosis. **A** Effect of G6PD expression level on the OS rate of patients with hepatocellular carcinoma in TCGA database. **B** Effect of G6PD expression level on PFS in patients with hepatocellular carcinoma in TCGA database. **C** Effect of G6PD expression level on the OS rate of patients with hepatocellular carcinoma in GEO (GSE14520) database. **D** Effect of G6PD expression level on the OS rate of patients with hepatocellular carcinoma in ICGC database

The Kaplan–Meier curve plotted had been described by the log-rank test, which showed that the high expression level of *G6PD* patients had poor OS compared with the low expression level of *G6PD* patients (*p* < 0.01, Fig. 3A). Meanwhile, In the PFS curve, the PFS of patients with a high expression level of *G6PD* was observably lower than that of patients with a low expression level (*p* < 0.01, Fig. 3B). The results of GEO database and ICGC database were consistent with the TCGA database (Fig. 3C-D). Overall Survival odds ratios (OR) for the TGCA, GEO, and ICGC databases, respectively 2.03 (1.46–2.83), 1.56 (1.01–2.41), 2.96 (1.50–5.86).

### Establishment and evaluation of the prognostic nomogram

**Fig. 4:**
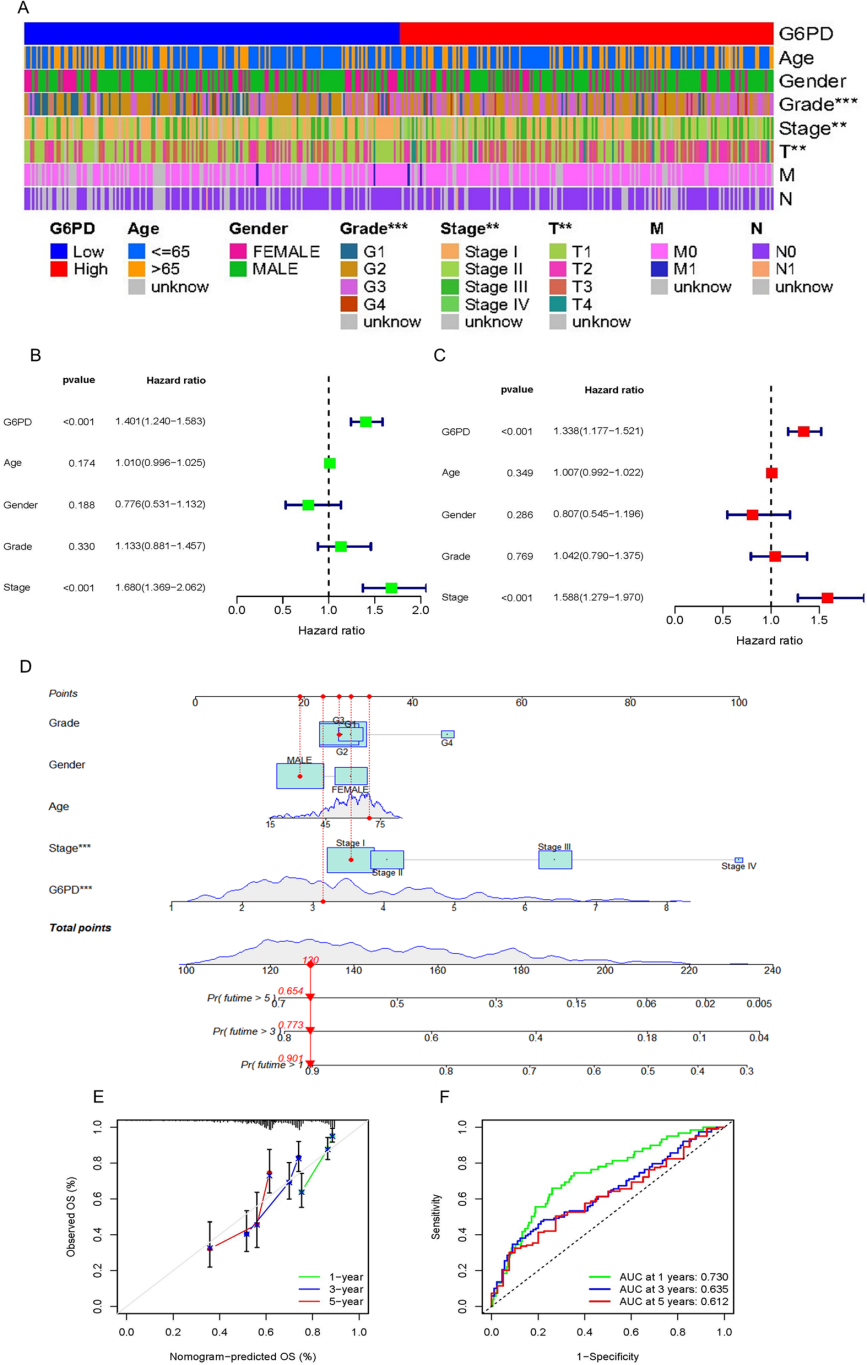
Prognostic Nomogram Establishment and Validation. **A** Heat map of the correlation between G6PD expression level and Age, Gender, Grade, Tumor stage, TNM stage. **B**-**C** Univariate and Multivariate Cox Regression. **D** Nomogram. **E** Calibration curves of nomogram on consistency between predicted and observed one-, three-, and five-year survival. **F** ROC curve analysis

First, we explored the relationship between *G6PD* and Age, Gender, Grade, Tumor stage and TNM stage, and found that *G6PD* expression level was dramatically different in Grade, Tumor stage and T stage, respectively (Fig. S2A-G). the heatmap was described, which brings together a variety of factors (Fig. 4A). To establish a prognostic nomogram consisting of multiple clinical features as a model for predicting the prognosis of LIHC, *G6PD*, Age, Gender, Grade, Tumor stage and TNM stage was taken into account. At the same time, univariate and multivariate Cox regression was carried out. According to univariate Cox regression, *G6PD* (*p* < 0.001), Stage (*p* < 0.001) and the difference in the T stage part were significant and statistically significant (Fig. 4B). As shown by multivariate analysis, the *G6PD* (HR = 1.338, 95% CI = 1.177–1.521, *p* < 0.001) and Tumor stage (HR = 1.588, 95% CI = 1.279–1.970, *p* < 0.001) were considered as the critical prognostic factors that forecasted the OS for LIHC (Fig. 4C). Then, we built a fresh nomogram to predict the one-, three-, and five-year OS rates of LIHC patients (Fig. 4D). Each patient corresponds to a score through the nomogram, and Patients with high scores had poorer outcomes than those with low scores. Survival calibration curves including one-, three- and five-year survival have been established. A scatter converging to a 45° diagonal would indicate a better model fit. The calibration curves show that our model predicts well (Fig. 4E). Finally, we evaluated the performance of the nomogram, and the results showed that the area under the curve (AUC) values of one-, three-, and five-year survival were 0.730, 0.635, and 0.612, respectively (Fig. 4F).

### Evaluation of the predictive power of Adaboost regression, Catboost regression and Gbdt regression

**Fig. 5:**
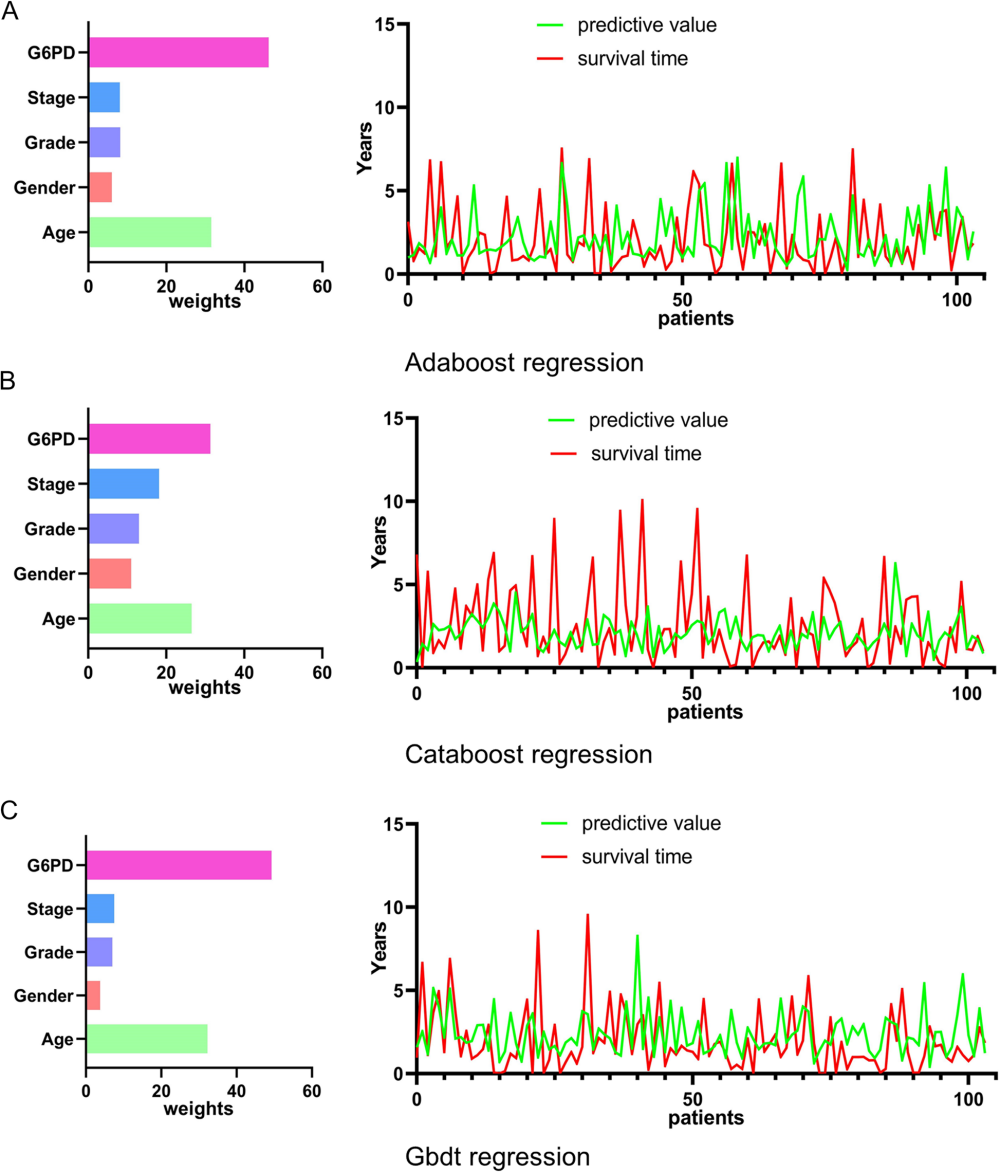
Variable importance plot and fitted curve from different Regression models. **A** Adaboost regression. **B** Catboost regression. **C** Gbdt regression

The results of Adaboost regression, Catboost Regression and Gbdt Regression showed that the prediction results fitted well with the actual value, and *G6PD* occupied a significant proportion of the clinical prognosis of LIHC. (Fig. 5A-C), The evaluation indexes of the three machine learning algorithms are shown in Table 1.

**Table 1:** Adaboost regression, Catboost regression, Gbdt regression statistical results

algorithms	indexes	Training set	Cross-validation set
Adaboost	MSE	0.036	5.323
	RMSE	0.189	2.281
	MAE	0.064	1.644
Catboost	MSE	0.329	4.361
	RMSE	0.574	2.058
	MAE	0.434	1.542
Gbdt	MSE	0.007	6.723
	RMSE	0.086	2.571
	MAE	0.041	1.963

### *G6PD* knockout inhibited the proliferation, migration and invasion of hepatocellular carcinoma cells

**Fig. 6:**
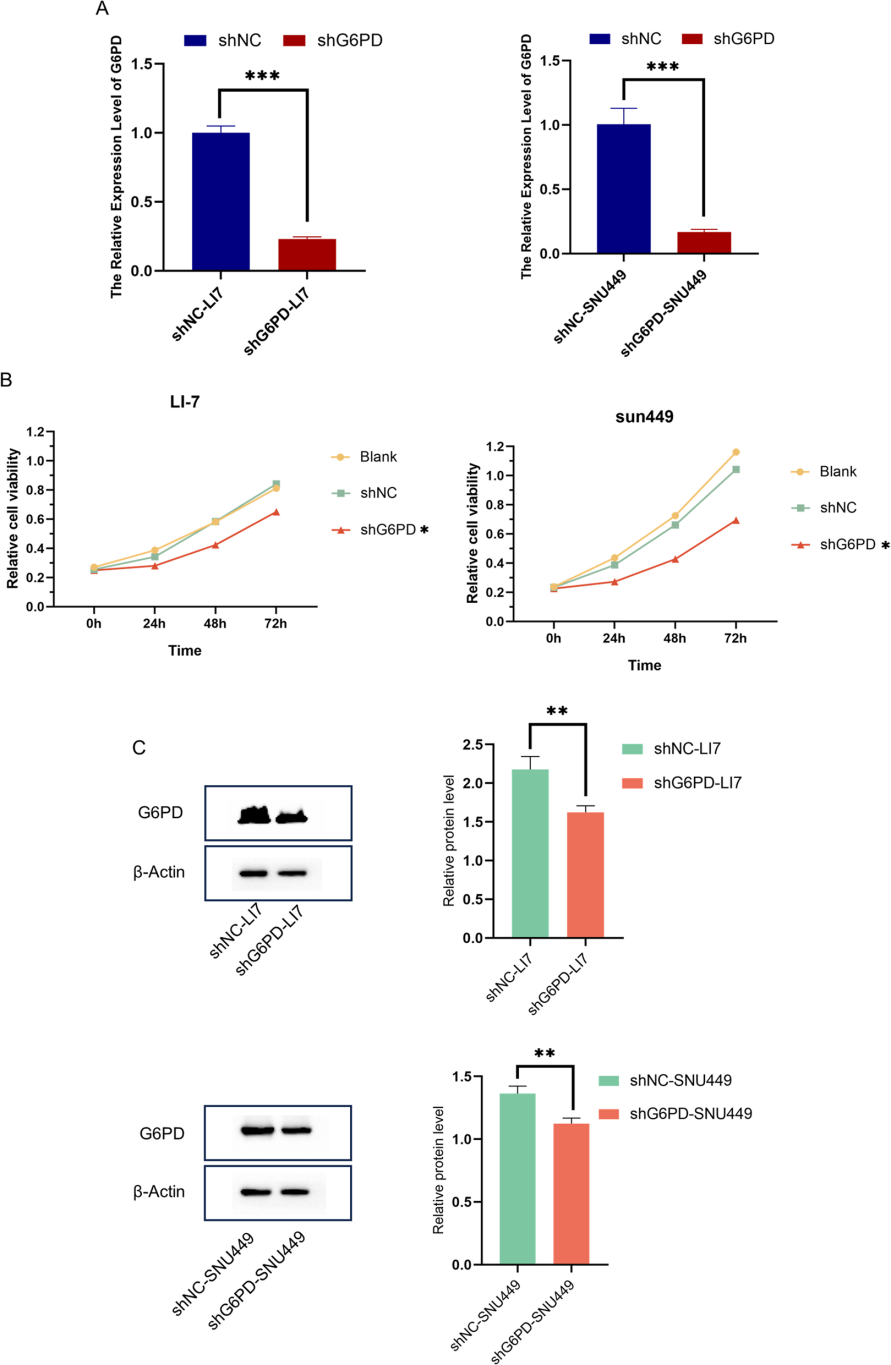
Effect of G6PD knockout on proliferation, migration and invasion of hepatocellular carcinoma cells. **A**, **C** PCR and WB results of G6PD expression in knockout cells. **B** Effect of G6PD knockout on cell proliferation. *P < 0.05, **P < 0.01, ***P < 0.001

To confirm the effect of *G6PD* on liver cancer cells, we transfected genOFF st-h-*G6PD* into LI-7 and SNU-449 liver cancer cells using transfection reagents. PCR experiments showed that *G6PD* gene was knocked down in both cells (Fig. 6A), and the results of western blots experiments were shown in Fig. (Fig. 6C). The cell proliferation experiment showed that the low expression of *G6PD* significantly inhibited the activity of SNU-449 and LI-7 cells, and the proliferation rate decreased significantly compared with normal and NC cells (Fig. 6B). Cell migration and invasion experiments showed that the migration and invasion ability of cells with low *G6PD* expression was weakened (Fig. S3A-B, Fig. S4A-B).

### Gene correlation analysis

**Fig. 7:**
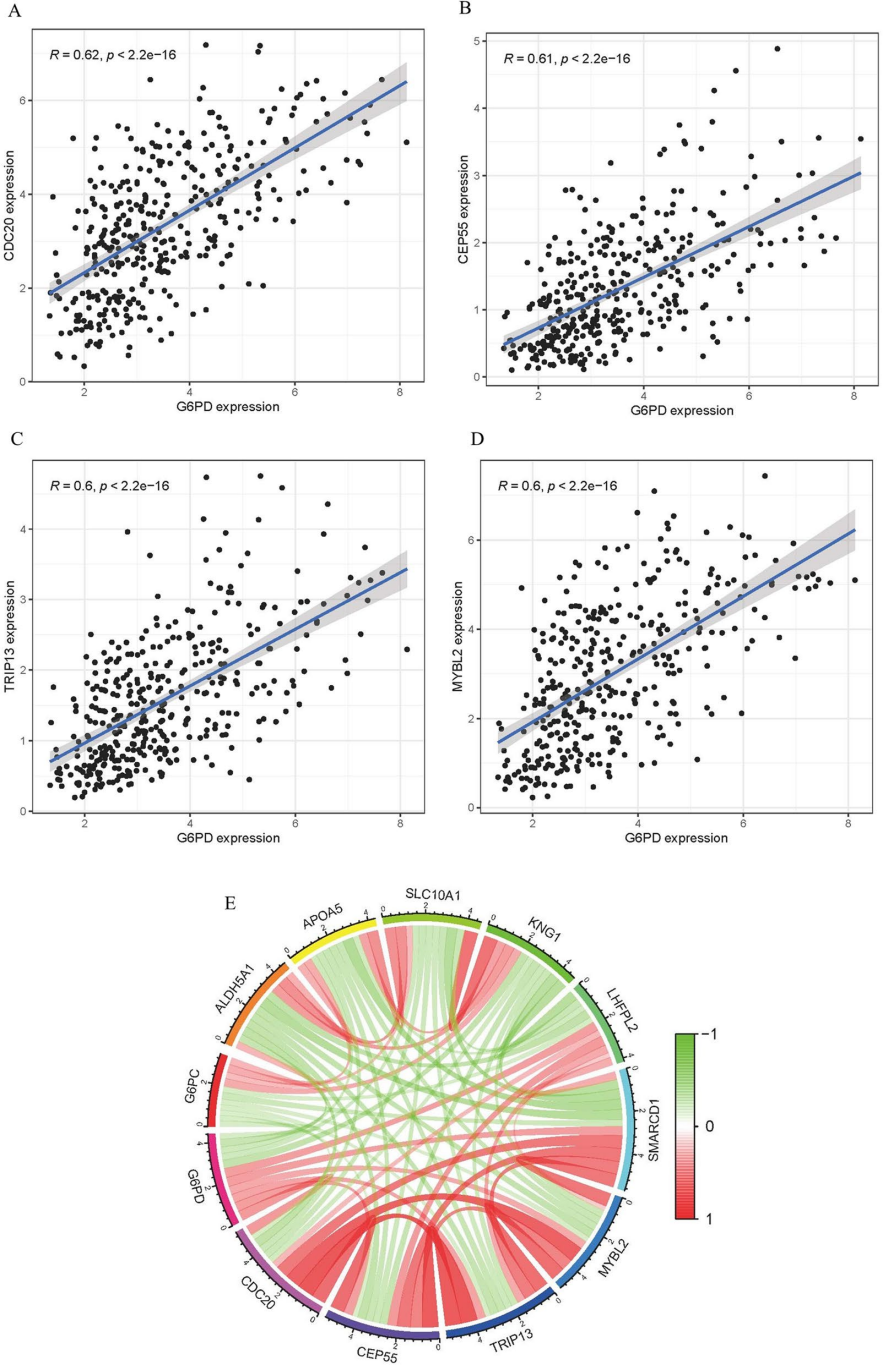
Gene Correlation Analysis. **A** Correlation of G6PD with CDC20 gene analyzed using TCGA database data. **B** Correlation of G6PD with CEP55 gene analyzed using TCGA database data. **C** Correlation of G6PD with TRIP13 gene analyzed using TCGA database data. **D** Correlation of G6PD with MYBL2 gene analyzed using TCGA database data. **E** Correlation of G6PD with SMARCD1, LHFPL2, KNG1, SLC10A1, APOA5, ALDH5A1, G6PC genes analyzed using TCGA database data

For investigating the correlation between *G6PD* and other genes, Correlation Analysis was performed by R package. *G6PD* was positively correlated with *CDC20, CEP55, TRIP13, MYBL2*, and the correlation was statistically significant, respectively, R = 0.62, 0.61, 0.6, 0.6 (Fig. 7A-E).

### GO, KEGG and GSEA analysis

**Fig. 8:**
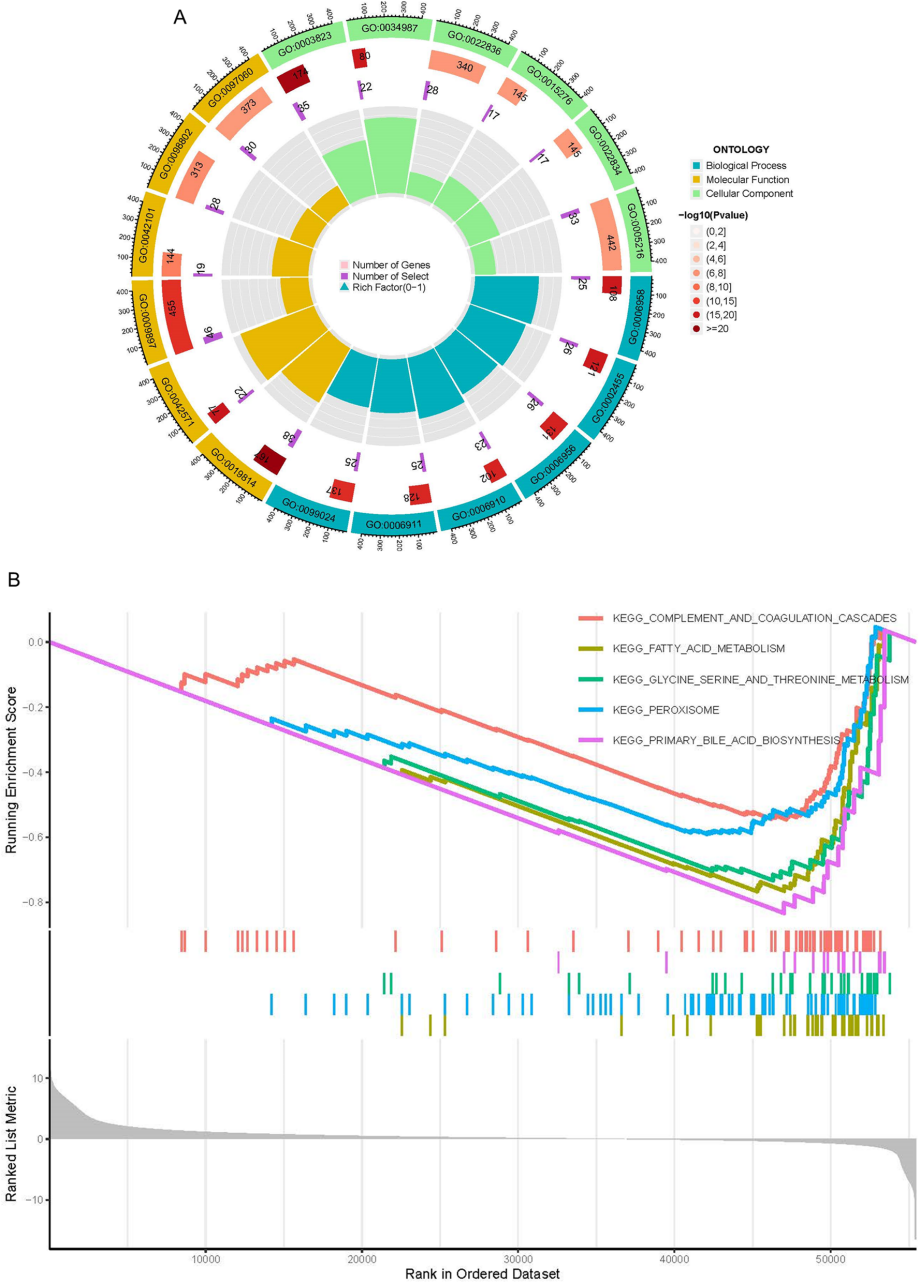
Functional Enrichment Analyses. **A** GO functional enrichment analysis of G6PD using TCGA database data. **B** GSEA enrichment analysis of G6PD using TCGA database data

GO, KEGG and GSEA enrichment analysis were implemented to comprehend the biological processes, cellular components, molecular function and related signaling pathways of *G6PD*. The GO analysis results showed that the biological processes of *G6PD* primarily assembled in immunoglobulin and B cell-mediated immune response, humoral immune response, immunoglobulin complement activation. The cellular component of *G6PD* mainly focused on external side of plasma membrane, neuronal cell body, synaptic membrane, and plasma membrane signaling receptor. The molecular function of *G6PD* mainly enriched in channel activity, antigen binding, passive transmembrane transporter activity and ion channel activity (Fig. S5A, Fig. 8A). The KEGG analysis results indicated that the signal pathway of *G6PD* mainly focused on Neuroactive ligand − receptor interaction pathway, Cell adhesion molecules pathway, PI3K − Akt signaling pathway and Cytokine − cytokine receptor interaction pathway (Fig. S5B). GSEA enrichment analysis shown that the pathway of the low expression group of *G6PD* was mainly enriched in Complement and Coagulation Cascades, Fatty Acid Metabolism, Glycine Serine and Threonine Metabolism, Peroxisome, Primary Bile Acid Biosynthesis (Fig. 8B).

### The connection between *G6PD* expression and immune system

**Fig. 9:**
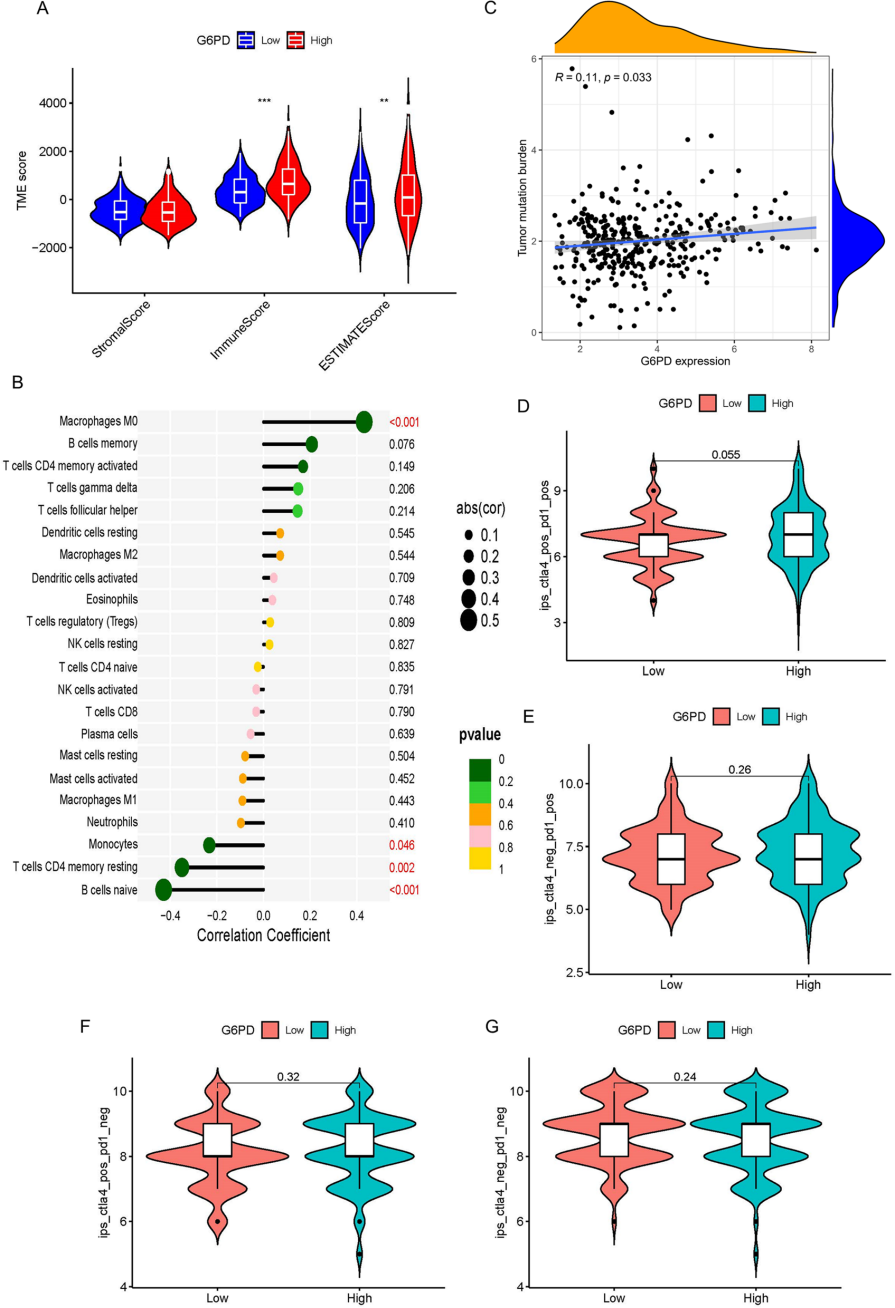
Immunity Analyses. **A** Effect of G6PD expression level on TME. **B** Relationship between G6PD expression level and immune cells. **C** Relationship between G6PD expression level and tumor mutation burden. **D**-**G** Value of risk score for immune checkpoint blockade. *P < 0.05, **P < 0.01, ***P < 0.001

To further research the connection between the expression level of *G6PD* in LIHC patients and immune status, the connection between *G6PD* and the TME score, immune cell, immune checkpoint was carried out. As shown, compared with the high expression level of *G6PD*, the TME score of immune score and the estimated score were lower in the low expression level of *G6PD*, and the difference was statistically significant, the difference in stromal cell score was not statistically significant (Fig. 9A). Analysis of immune cell subsets suggested that immune cell scores, including B cells naïve, Macrophages M0, Monocytes, T cells CD4 memory resting were markedly different between the low- and high-expression level of *G6PD* groups, respectively, R = -0.43, 0.43, -0.23, -0.35, *P* < 0.05 (Fig. 9B, Fig. S6A-E). Then, Fig. 9C showed that the relationship between *G6PD* and tumor mutation burden, R = 0.11, *p* < 0.05. this indicated that LIHC patients with high *G6PD* expression levels had high TMB scores and good immunotherapy effects. Fig. S6F showed the coefficient diagram of a relationship between *G6PD* and immune checkpoint. At the same time, we analyzed routine immune checkpoints including PD1 and CTLA4. Whether the expression level of *G6PD* was high or low, the mean IPS showed no significance. (Fig. 9D-G).

### Drug sensitivity analysis

**Fig. 10:**
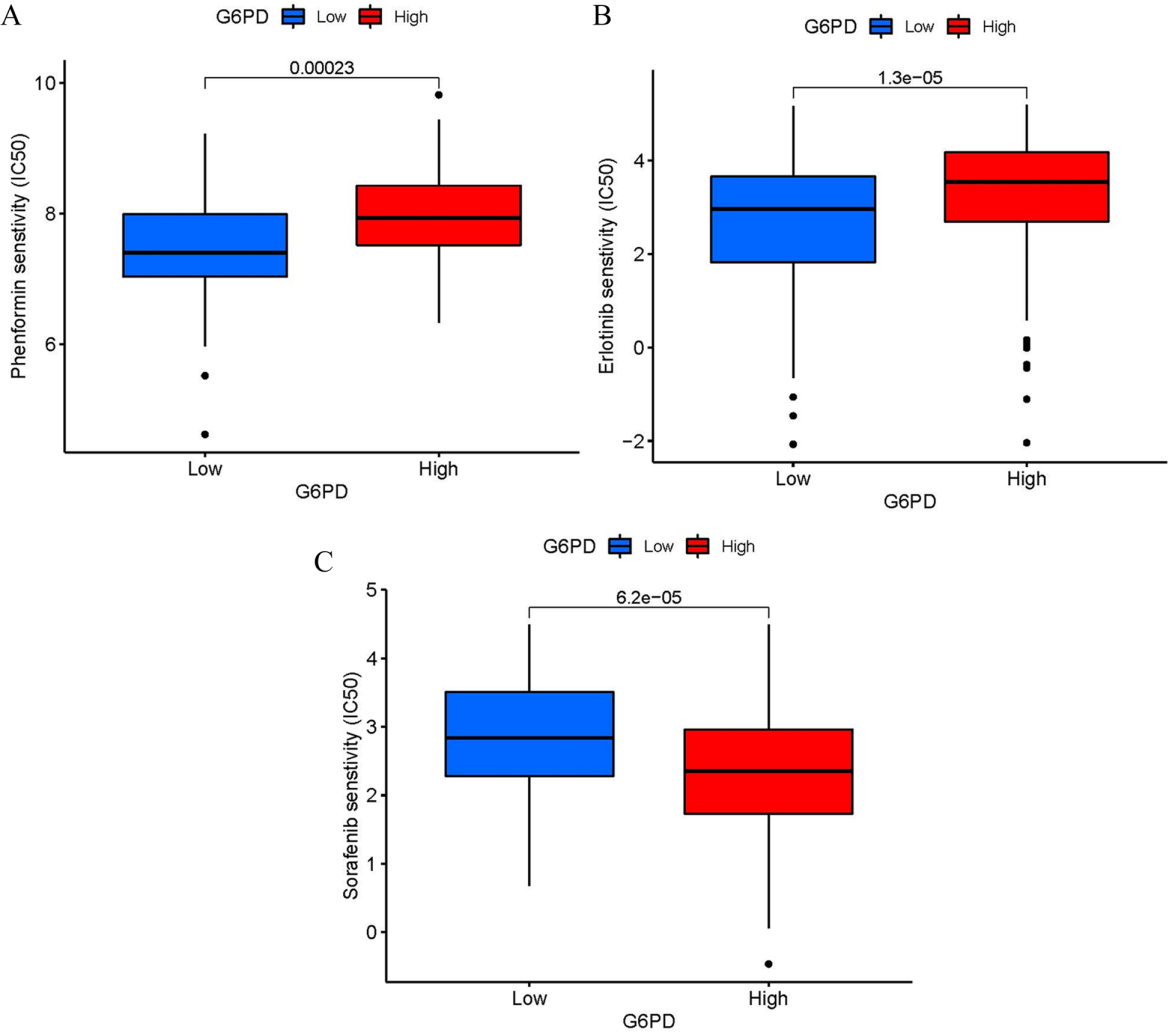
Drug sensitivity analysis. **A**-**B** Phenformin and Erlotinib were sensitive in the G6PD high expression group. **C** Sorafenib was sensitive in the G6PD high expression group

To explore the clinical significance of *G6PD*, the drug sensitivity of LIHC was predicted by R software. The consequences revealed that the high expression of the *G6PD* group was more sensitive to 2 kinds of drugs, including Phenformin and Erlotinib (Fig. 10A-B). In comparison, the low expression of the *G6PD* group was more sensitive to Sorafenib (Fig. 10C).

### Prediction of clinical prognosis of LIHC patients by four machine learning methods

**Fig. 11:**
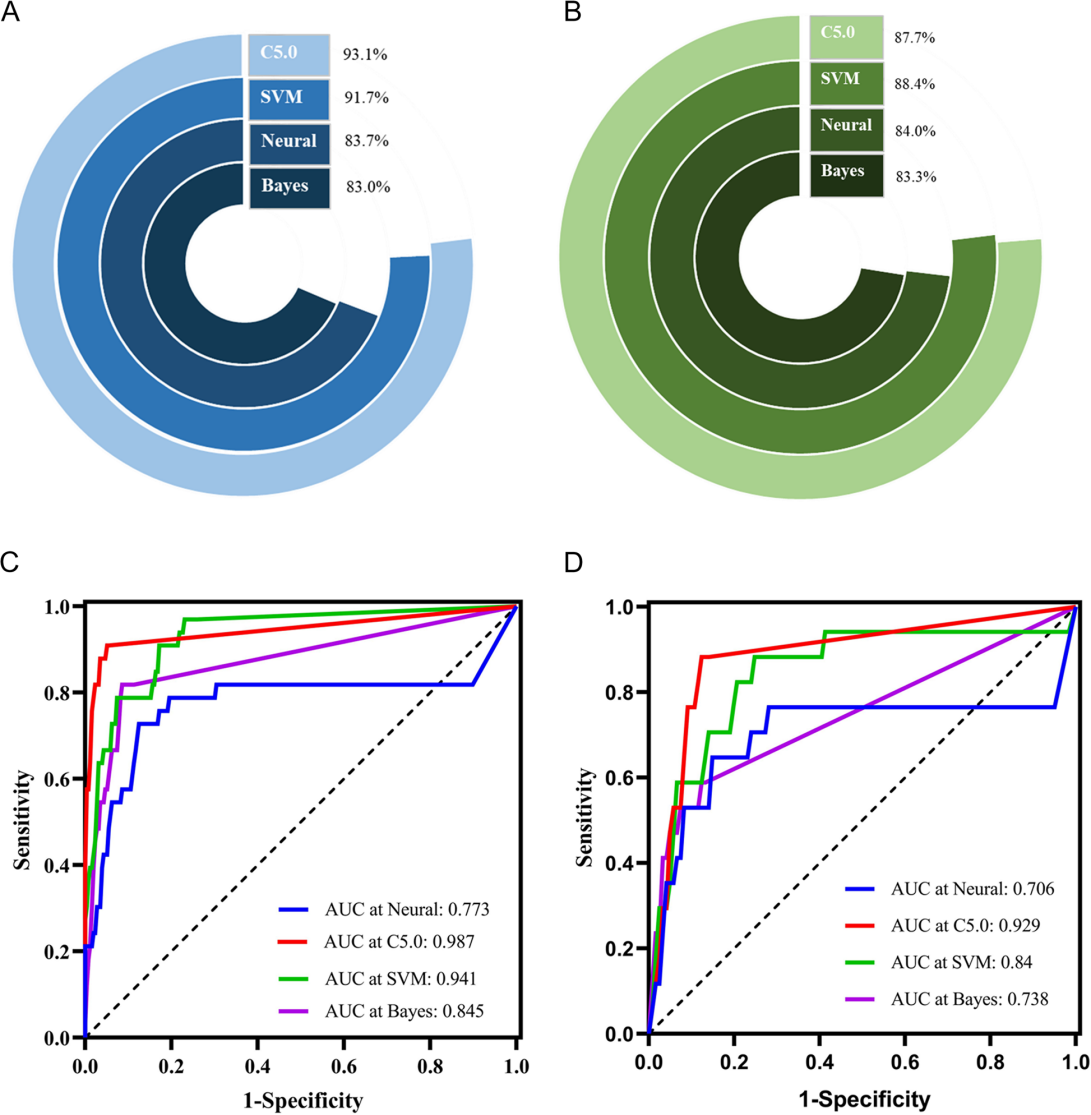
Prediction of clinical prognosis of hepatocellular carcinoma by Four Machine learning methods including Bayesian Classifier, Neural network algorithm, Support Vector Machine and Decision Tree C5.0. **A**-**B** The correct rate of four machine algorithms in the training group and Testing group. **C**-**D** The ROC curve of evaluating the prediction of the four algorithms

To predict the clinical prognosis of patients with LIHC, we selected four machine learning algorithms, including the Bayesian Classifier, Neural network algorithm, Support vectors machine, and Decision Tree C5.0. The research results showed that in the training group, the correct rates of the Bayesian Classifier, Neural network algorithm, Support vectors machine, and Decision Tree C5.0 were 83.04%,83.74%, 91.70%, and 93.08%, respectively (Fig. 11A). In the test group, the correct rates were 83.33% 84.06%, 88.41% and 87.68%, respectively. (Fig. 11B). At the same time, we evaluated the prediction performance of the four algorithms, and the evaluation outcomes revealed that in the training group, the AUC of Bayesian Classifier, Neural network algorithm, Support vectors machine and Decision Tree C5.0 were 0.845,0.773,0.941,0.987, respectively (Fig. 11C). In the test group, the AUC were 0.738,0.706,0.84 and 0.929, respectively (Fig. 11D).

## Discussion

Baba et al*.* found that the expression levels of *G6PD* may be strongly associated with the growth of precancerous and neoplastic lesions [9]. Dore et al*.* found that *G6PD* deficiency significantly reduced the incidence of hepatocellular carcinoma in a case–control study [16]. However, the mechanism between *G6PD* expression and LIHC remains unclear. In this study, we explored the correlation between *G6PD* and LIHC, and the possible signaling pathways leading to LIHC by bioinformatics analysis. We found a statistically prominent difference in *G6PD* expression level between cancer tissues and para-carcinoma tissues of patients with LIHC. Immunohistochemical results obtained from the human protein atlas confirmed that *G6PD* expression levels were significantly increased in LIHC. We also confirmed this by PCR and WB experiments on one type of normal liver cells and four types of liver cancer cells.

Tumor mutation burden has long been used as a biomarker for tumor prediction [17]. Consequently, we investigated the tumor mutation burden of *G6PD*, and its prognostic impact on hepatocellular carcinoma and evaluated the plausibility of the impact. The expression levels of *G6PD* were positively related to the tumor mutation burden, indicating that the group with high G6PD expression levels had a poor immunotherapy outcome and a poor prognosis, which was the same as the research result of Cao [18]. We also found that elevated *G6PD* expression resulted in poor OS and PFS in LIHC. We studied the influence of *G6PD* on the proliferation, migration and invasion of LI-7 and SNU-449 by knockout of *G6PD* gene. The results showed that *G6PD* knockdown significantly reduced the proliferation rate, migration and invasion ability of LI-7 and SNU-449. Cao et al. showed that the up-regulation of *G6PD* promoted the survival, metastasis, and invasion of HepG2 cells [18]. Lu et al*.* showed that the high expression of *G6PD* was significantly connected to the metastasis and poor prognosis of LIHC, and the migration and invasion of LIHC cells were inhibited when the *G6PD* gene was knocked out [19]. Li et al*.* showed that the low expression of *G6PD* observably prolonged the orthotopic tumor model mice's survival time. When the high expression of *G6PD* resumed, the tumor growth, tumor size, volume, and weight were restored [20]. High expression of G6PD produces high levels of NADPH via the PPP pathway, as well as an increase in glutathione, which in turn counteracts oxidative stress and DNA damage, which promotes immune escape, tumor progression, and drug resistance [5]. Therefore, high G6PD expression is closely associated with poor LIHC prognosis, and high G6PD expression promotes the migration and invasive ability of LIHC cells through reorganization of the glucose metabolism pathway.

We found a positive connection between the expression level of *G6PD* and *CDC20, CEP55, TRIP13, MYBL2* in LIHC patients. Several scientists pointed out that *CDC20, CEP55, TRIP13, MYBL2* were overexpressed in hepatocellular carcinoma, compared with adjacent normal tissues [21–24]. However, their interactions in promoting hepatocellular carcinoma are not investigated. GO, KEGG and GSEA analysis was executed to probe the possible involvement of *G6PD* in hepatocellular carcinoma formation. Our study showed that *G6PD* was mainly involved in the immune system and signaling pathway (e.g., PI3K-Akt signaling pathway, Cell adhesion molecules pathway) in promoting the occurrence and development of LIHC. Cheng et al*.* indicated that there was an interaction between the PI3K-Akt signaling pathway and *G6PD*, which promoted the development of cancer [25].

By immune analysis, we discovered that the expression level of *G6PD* was positively related to Macrophages M0, and negatively correlated with B cells naive, Monocytes, and T cells CD4 memory resting. Tekin et al*.* showed that macrophage M0 had anti-tumorigenic activity and impaired the growth of pancreatic cancer cells through TNF-α secretion [26]. Therefore, the increase of macrophage M0 may have an inhibitory effect on LIHC. Nevertheless, there is no research on the correlation between Macrophages M0, B cells naive, Monocytes, T cells CD4 memory resting and hepatocellular carcinoma. Immune checkpoint analysis showed that *G6PD* was positively associated with most immune checkpoints. Still, the results of the risk score values of immune checkpoint blockade (ICB) suggested that *G6PD* may not played a role in predicting the risk score model of PD1 and CTLA4 treatment response.

The drug sensitivity test found that the group with high *G6PD* expression level was sensitive to Phenformin and Erlotinib, and the group with low *G6PD* expression level was sensitive to Sorafenib. Phenformin is a diabetes treatment but causes a fatal lactic acidosis reaction [27]. Recently, a study has suggested that Phenformin has an anti-tumor effect and can inhibit the glucose metabolism of tumor cells [28]. Huang et al*.* found that the combination of Phenformin and Sorafenib showed a synergistic ability to restrain the proliferation and migration of LIHC [29]. Erlotinib is a treatment for non-small cell lung cancer [30]. Zheng et al*.* indicated that the efficacy of Erlotinib in the treatment of hepatocellular carcinoma was unclear, but they found that 2-methoxy estradiol enhanced the inhibitory effect of Erlotinib on hepatocellular carcinoma [31]. Phase 3 clinical trial showed that the combination of sorafenib and erlotinib had little effect on the survival rate of patients with advanced LIHC [32]. Sorafenib is the gold standard for the treatment of advanced hepatocellular carcinoma. However, due to individual heterogeneity, the resistance to Sorafenib has gradually attracted attention [33]. High glucose metabolism due to GLUT1/ALDOB/G6PD axis expression promotes drug resistance in pancreatic cancer, and inhibition of the GLUT1/ALDOB/G6PD axis may serve as a target for drug resistance therapy [34]. Thus, the high level of *G6PD* may be an important reason for Sorafenib resistance.

The Bayesian classifier is an artificial intelligence widely used in medical decision-making. Junath et al. applied it to the prognosis diagnosis of breast cancer and considered it feasible and effective [35]. Bo et al*.* believed that using support vector machines could effectively predict the time and location of cancer recurrence, and the effect was better than that of the neural network algorithm, which was consistent with our research results [36]. Noh et al*.* trained the Decision Tree C5.0 decision tree classifier by adding 7 histological features to predict the prognosis of patients with advanced gastric cancer and concluded that the model had a more accurate prediction effect [37]. In this study, TCGA database data was used to compare the performance of these four machine-learning algorithms. We believe that Decision Tree c5.0 can predict the prognosis of LIHC patients better than other algorithms.

Of course, there are still many shortcomings in this study. First, the data in this study come from a public database, and we don’t supervise the collection of data, so the reliability of the data is not clear. Second, the research results are only obtained through data analysis, and the credibility of the results needs to be demonstrated through experimental research.

## Conclusion

In this investigation, we conducted a comprehensive assessment of *G6PD*'s clinical value as a diagnostic and prognostic indicator for LIHC. Our study results demonstrated the potential of *G6PD* to enhance LIHC diagnosis, enabling early detection and prompt treatment for affected patients. Through meticulous cell experiments, we confirmed the substantial impact of low *G6PD* expression on the proliferative activity, migration, and invasion of liver cancer cells. Machine learning shows Decision Tree c5.0 has a better ability to predict the clinical prognosis of LIHC patients. In conclusion, *G6PD*, along with Decision Tree c5.0, holds promise as a valuable tool for predicting the prognosis of LIHC patients and offering diagnostic insights to clinicians.

### Supplementary Information

**Additional file 1.**

## Data Availability

The datasets analyzed during the current study are available from the Cancer Genome Atlas (TCGA) database (https://portal.gdc.cancer.gov/projects/TCGA-LIHC, dbGaP Study Accession:phs000178), the International Cancer Genome Consortium (ICGC-LIRI-JP cohort, https://dcc.icgc.org/projects/LIRI-JP) database and the Gene Expression Omnibus (GEO: GSE14520, GSE20140, GSE62232, GSE84005) database (http://www.ncbi.nlm.nih.gov/geo/).

## Abbreviations

LIHC: Liver Hepatocellular carcinoma
*G6PD*: Glucose-6-phosphate Dehydrogenase gene
PPP: The pentose phosphate pathway
NADPH: Nicotinamide adenine dinucleotide phosphate
TCGA: The Cancer Genome Atlas database
ICGC-LIRI-JP cohort: International Cancer Genome Consortium database
GEO: The Gene Expression Omnibus database
IHC: Immunohistochemistry
HPA: The protein atlas
OS: Overall survival
PFS: Progression-free survival
TMB: Tumor mutation load
ROC: The receiver operating characteristic curve
CDC20: Homo sapiens cell division cycle 20 gene
CEP55: Centrosomal protein 55kda gene
TRIP13: Homo sapiens thyroid hormone receptor interactor 13 *gene*
MYBL2: Homo sapiens v-myb avian myeloblastosis viral oncogene homolog-like 2
GO: The Gene Ontology
KEGG: Kyoto Encyclopedia of Genes and Genomes pathway analysis
GSEA: Gene-set enrichment analysis
IC50: Half-maximal inhibitory concentration
TCIA: The Cancer Immunoomics Atlas
HR: Hazard ratio
AUC: The area under the curve
ICB: Immune checkpoint blockade
PD1: Programmed cell death protein 1
CTLA4: Cytotoxic T-lymphocyte-associated protein 4
TME: Tumor microenvironment
