# Supplementary material for: G6PD and machine learning algorithms as prognostic and diagnostic indicators of liver hepatocellular carcinoma
Source: BMC Cancer. 2024 Jan 31;24:157. doi: 10.1186/s12885-024-11887-6 (PMC10829225; doi:10.1186/s12885-024-11887-6)

**Supplementary Methods**

**Methods**

**Calculation of tumor mutational burden**

Tumor mutation load calculation method: first we renamed the target file as "TMB.TXT" file, then read the file and the gene expression data file, deleted the normal and disease-free samples, combined the two data, defined the genome length or the number of genes covered, counted the number of mutations, which was used to calculate the TMB, and then performed a correlation analysis.

**Calculation steps of Nomogram**

Nomogram is a graphical tool for predicting the probability of an event and is commonly used in medical research, especially in clinical settings for predicting disease risk, probability of illness, or probability of survival. Nomogram accomplishes this by mapping the scores of multiple predictors to a total score, and mapping the total score to the probability of a particular event. Nomogram calculation steps: first we converted the data to "CSV" format and selected the features for prediction (age, gender, grading, TNM stage, G6PD); then we selected the target variables, extracted the features and target variables, and then we divided the training set and the test set, trained the logistic regression model, predicted the probability, and calculated the calibration curve and ROC curve.

**Selecting the basics of Adaboost regression, Catboost regression and Gbdt regression**

We chose models that could perform deep learning on the data based on the purpose and needs of the study, i.e., regression to study how well the predicted data fit the actual data and thus assess the predictive effect of the model, and to study the weight of G6PD and clinical features in causing LIHC. Adaboost regression, Catboost regression, and Gbdt regression can be used for modeling that contains LIHC patient-related biomarkers, gene expression data, or other clinical characteristics to make relevant predictions.

**GO, KEGG and GSEA Analysis**

we used GO enrichment analysis version: July, 2023; KEGG version: 107.1, August, 2023; GASE calculations were analyzed by the "GSEA.R" script in R software.

**Selecting the basics of Bayesian Classifier, Neural Network Algorithm, Support Vector Machine (SVM) and Decision Tree C5.0**

The selection of the model is based on the purpose and need of the study. The purpose of our study is to use the model to classify and identify the prognosis of LIHC patients, and to learn by training and then obtain the prediction of LIHC prognosis. Meanwhile Bayesian Classifier, Neural Network Algorithm, Support Vector Machine (SVM) and Decision Tree C5.0 have the ability to handle high dimensional data with potential feature learning ability to extract the most predictive features from the data. Data splitting principle: We randomly split the data into two disjoint training and validation groups to ensure that the distribution of the two groups is representative of the original data.

**Supplementary legends**

**
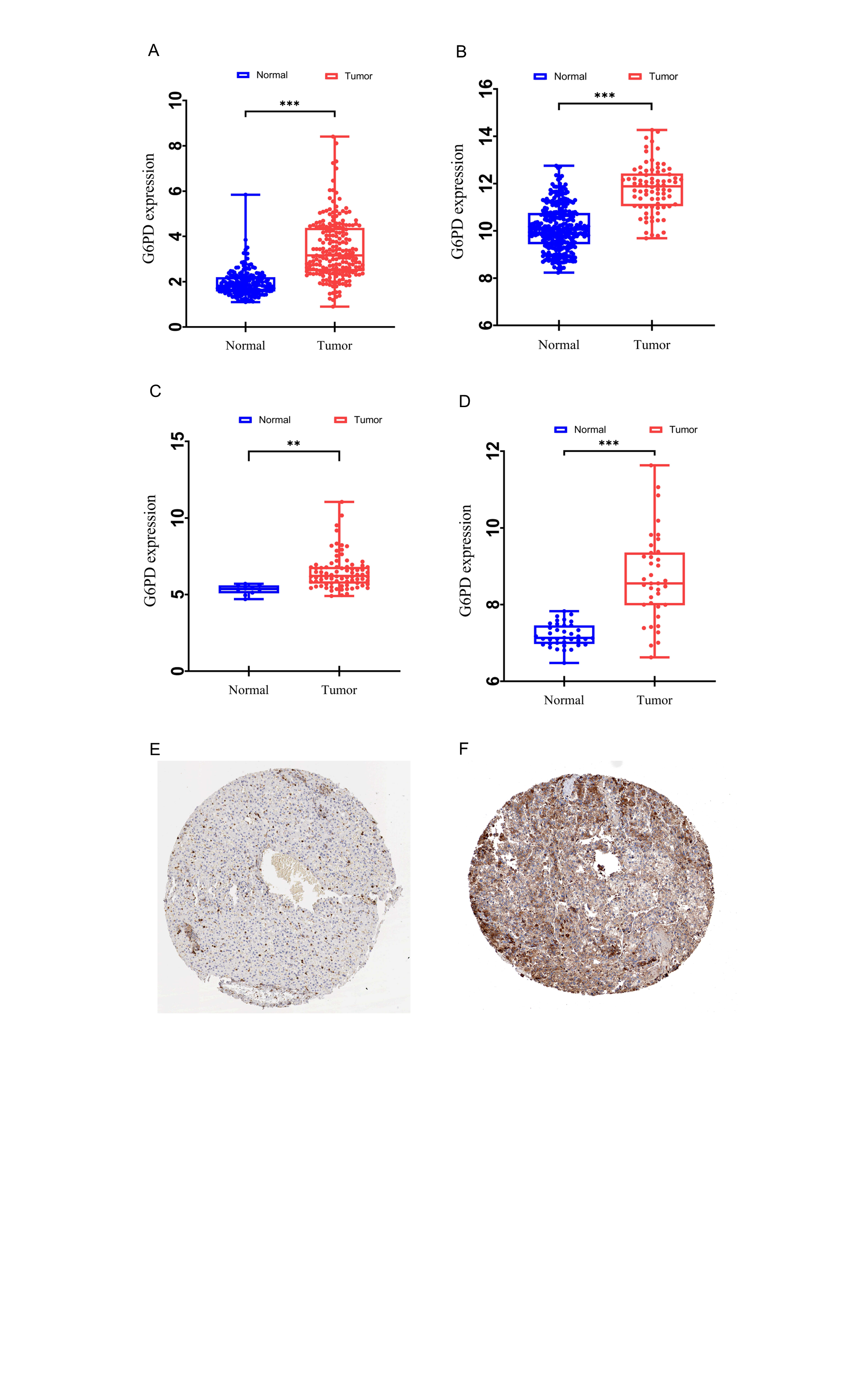
**

**Fig. S1** The Expression level of *G6PD*. (A-D) The different expression level of *G6PD* between normal and hepatocellular carcinoma tissues in ICGC, GSE20140, GSE62232, GSE84005, respectively. (E-F) Protein expression of *G6PD* in normal(E) and hepatocellular carcinoma tissues(F) in HPA database. *P < 0.05, **P < 0.01, ***P < 0.001.

**
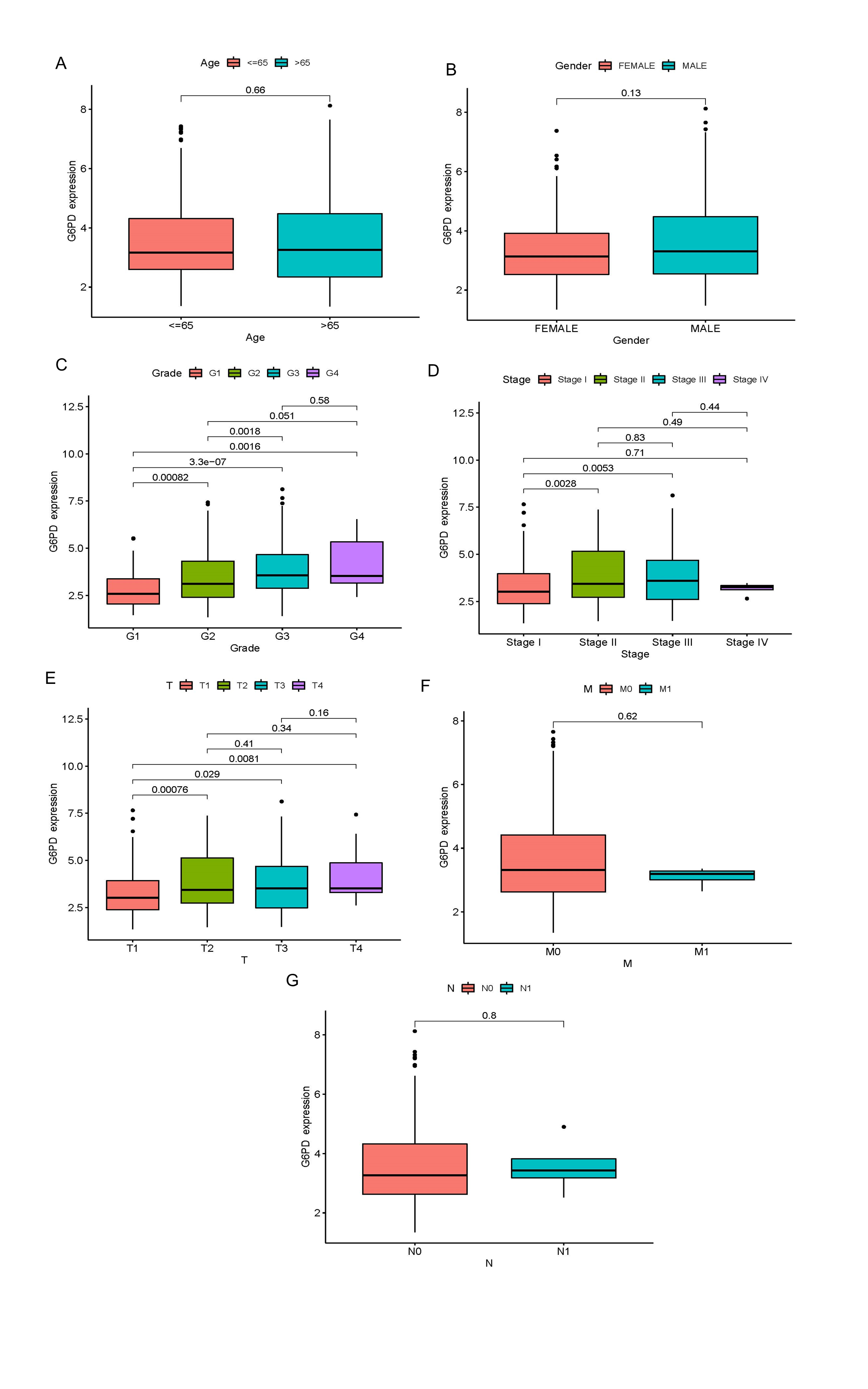
**

**Fig. S2** Prognostic Nomogram Establishment and Validation. (A-G) Relationship between *G6PD* expression level and Age, Gender, Grade, Tumor stage and TNM stage.

**
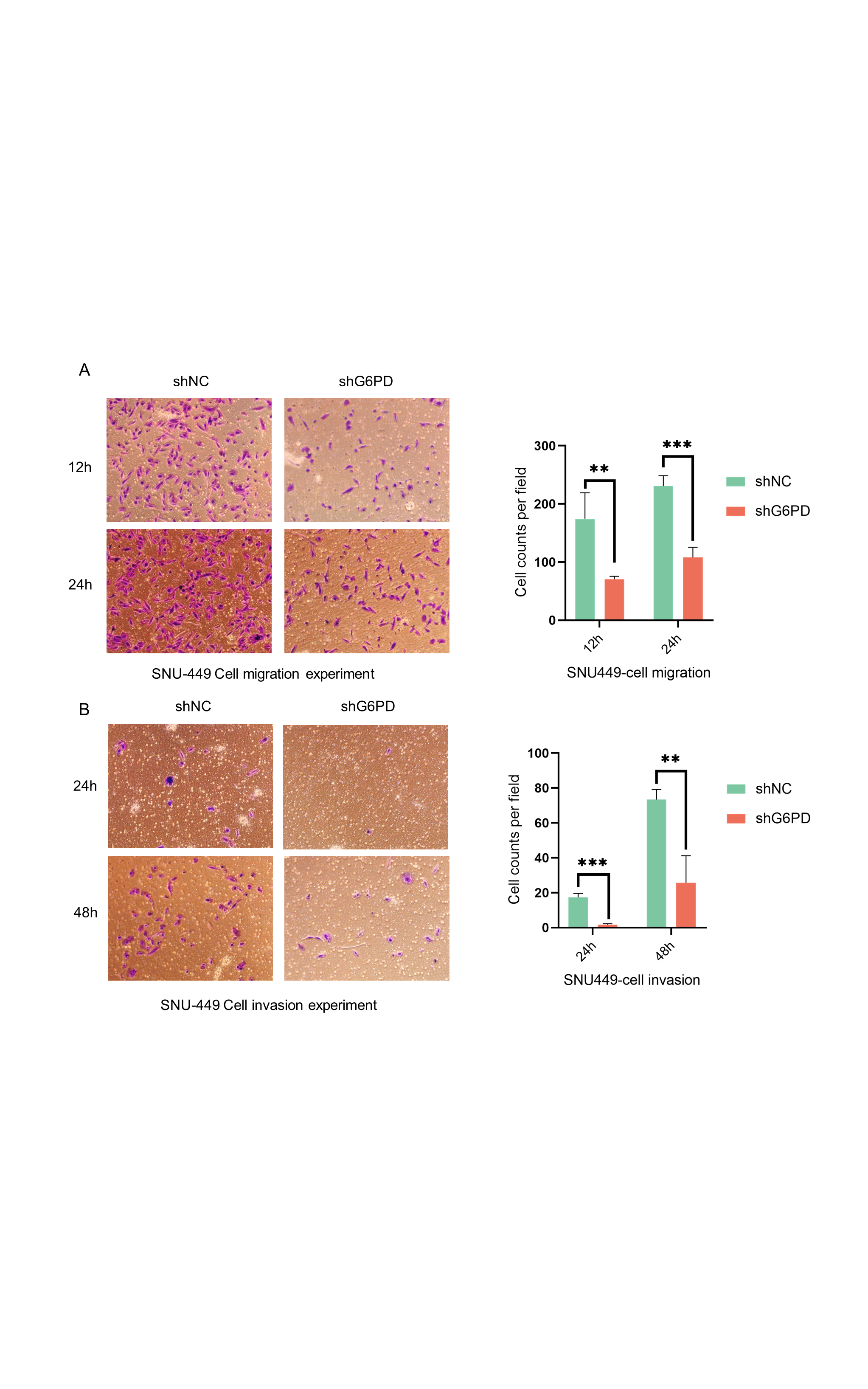
**

**Fig. S3** Effect of G6PD knockout on proliferation, migration and invasion of hepatocellular carcinoma cells. (A-B) Effect of G6PD knockout on migration and invasion of SUN449 cells. *P < 0.05, **P < 0.01, ***P < 0.001.

**
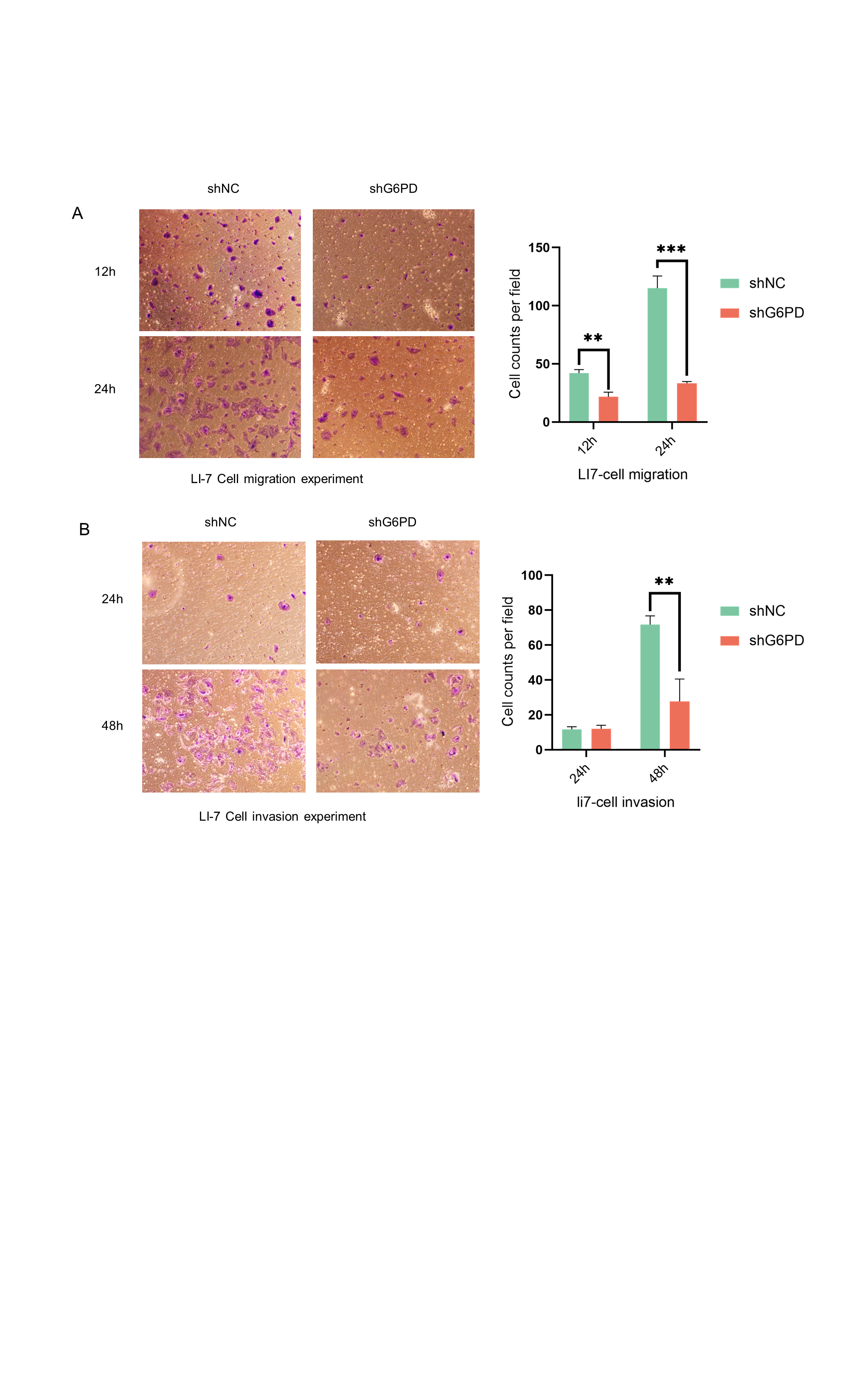
**

**Fig. S4**Effect of *G6PD* knockout on migration and invasion of hepatocellular carcinoma cells. (A-B) Effect of *G6PD* knockout on migration and invasion of LI7 cells. *P < 0.05, **P < 0.01, ***P < 0.001.

**
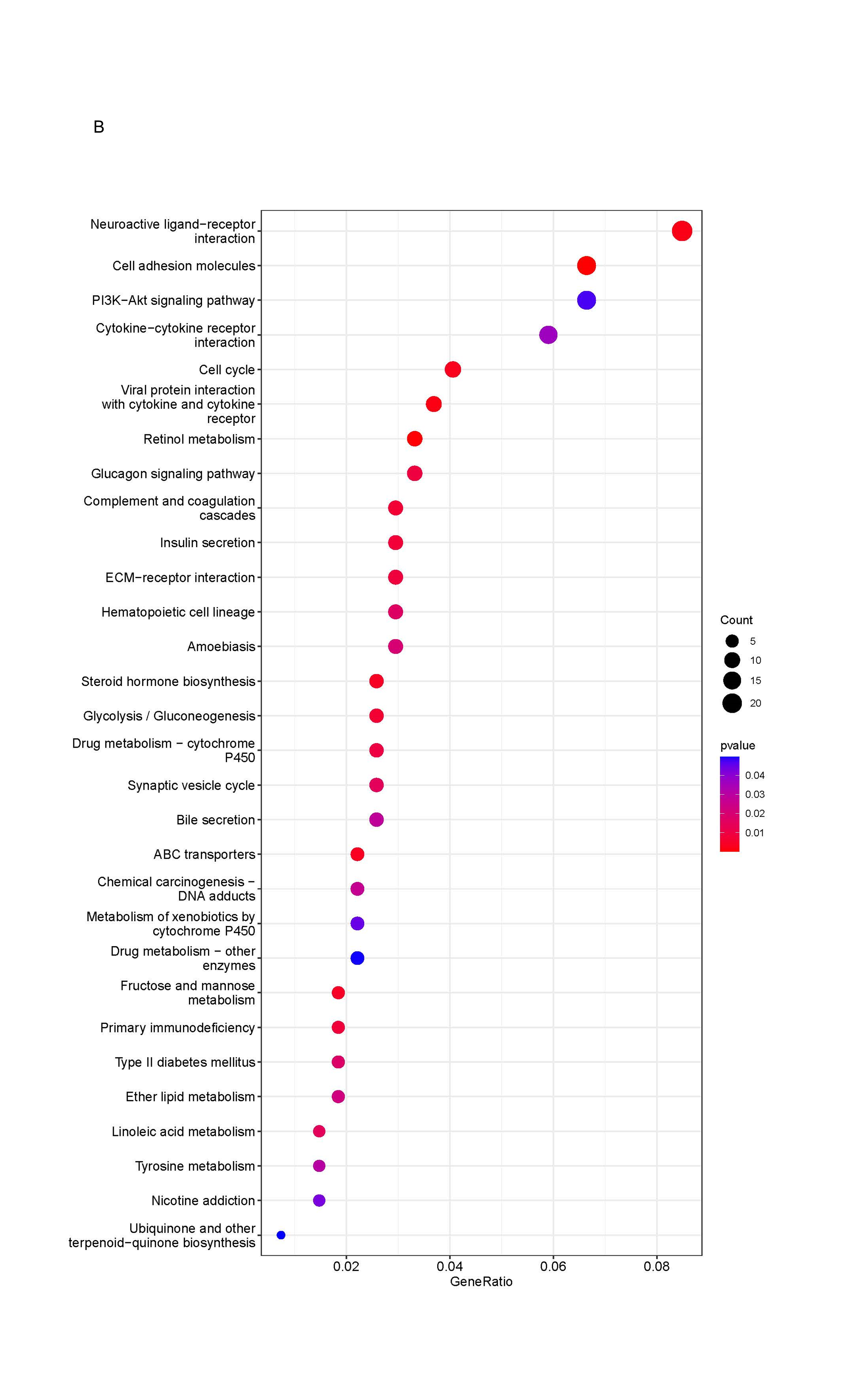

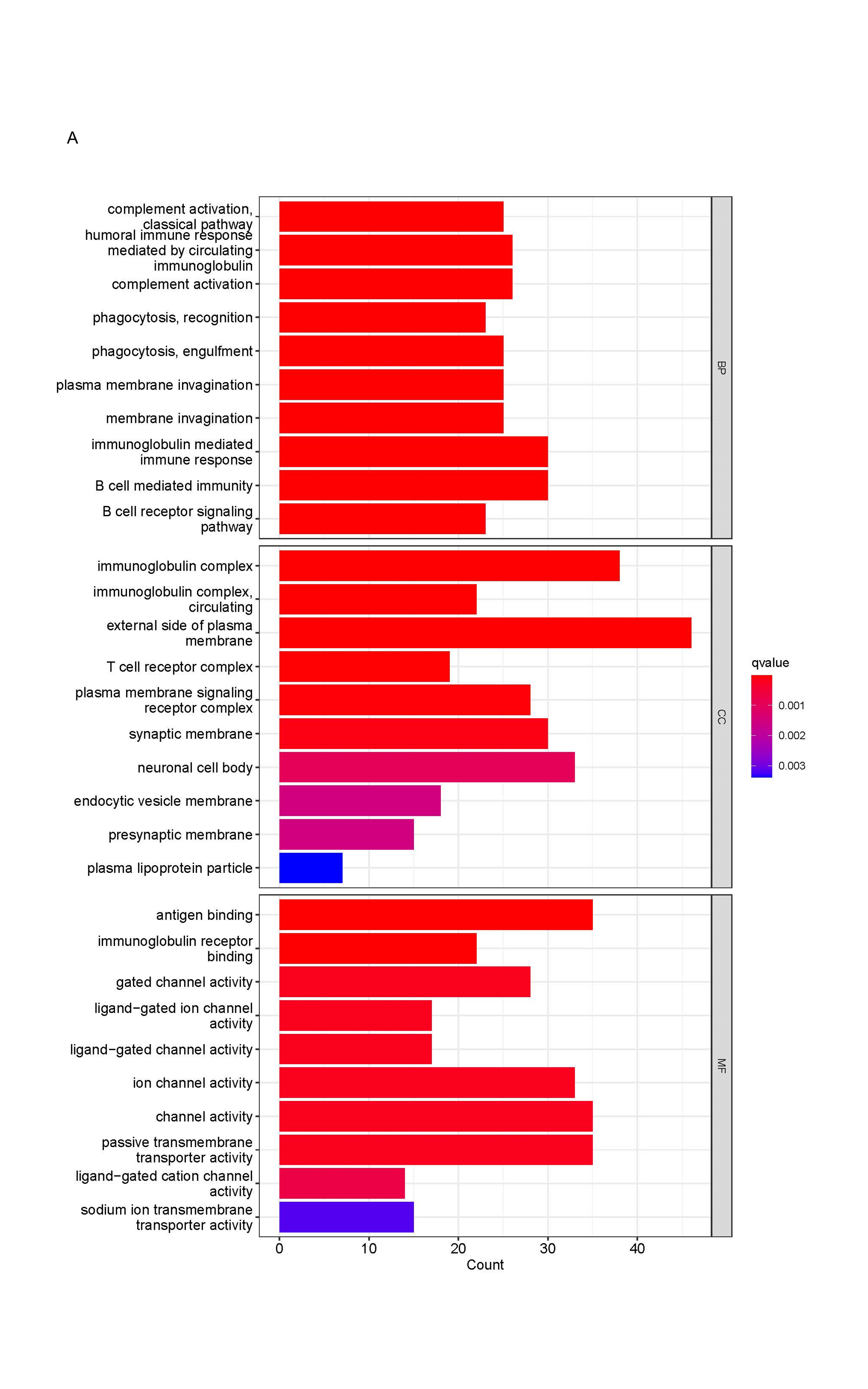
**

**Fig. S5** Functional Enrichment Analyses. (A) GO functional enrichment analysis of G6PD using TCGA database data. (B) KEGG pathway enrichment analysis of G6PD using TCGA database data. *P < 0.05, **P < 0.01, ***P < 0.001.

**
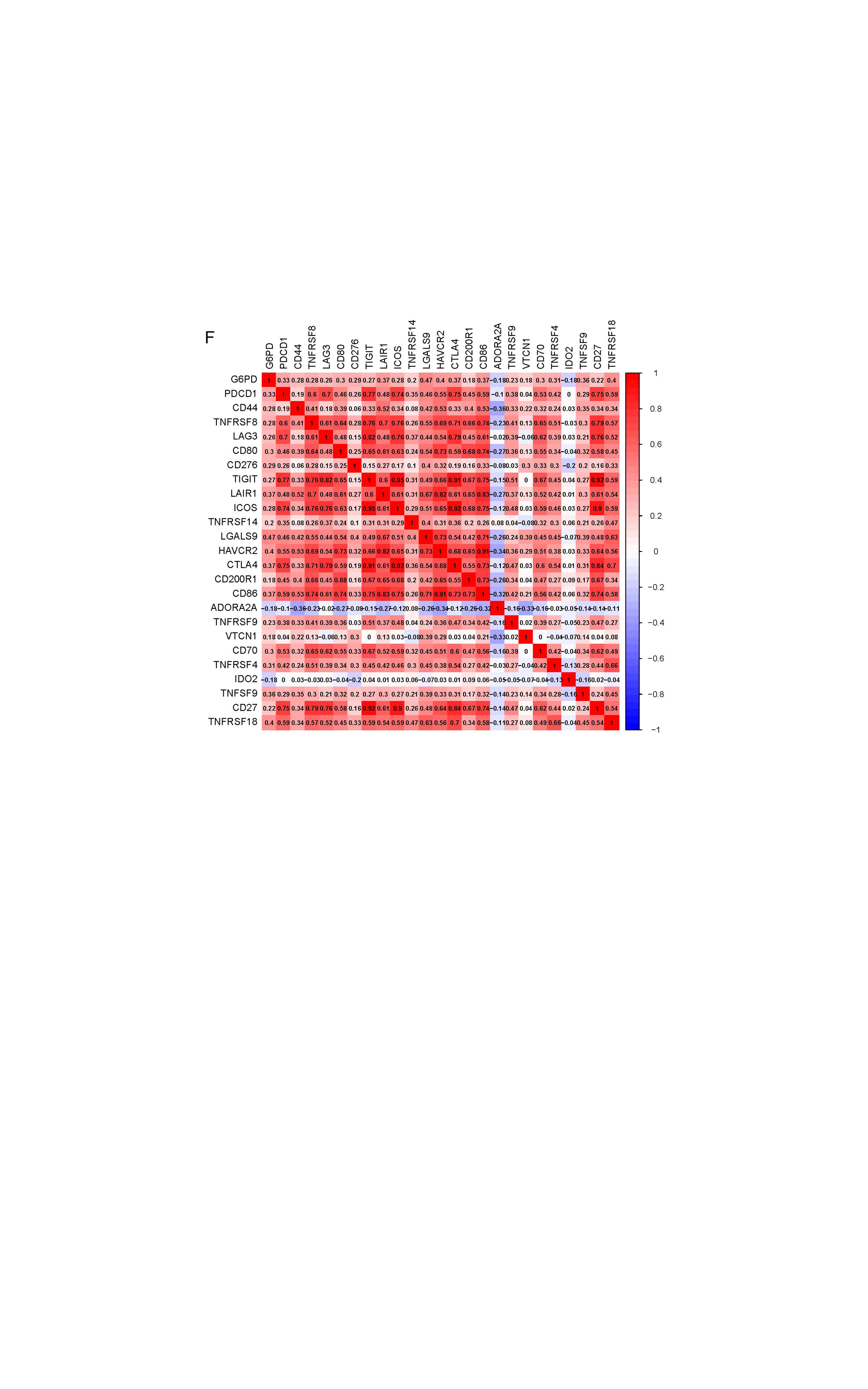

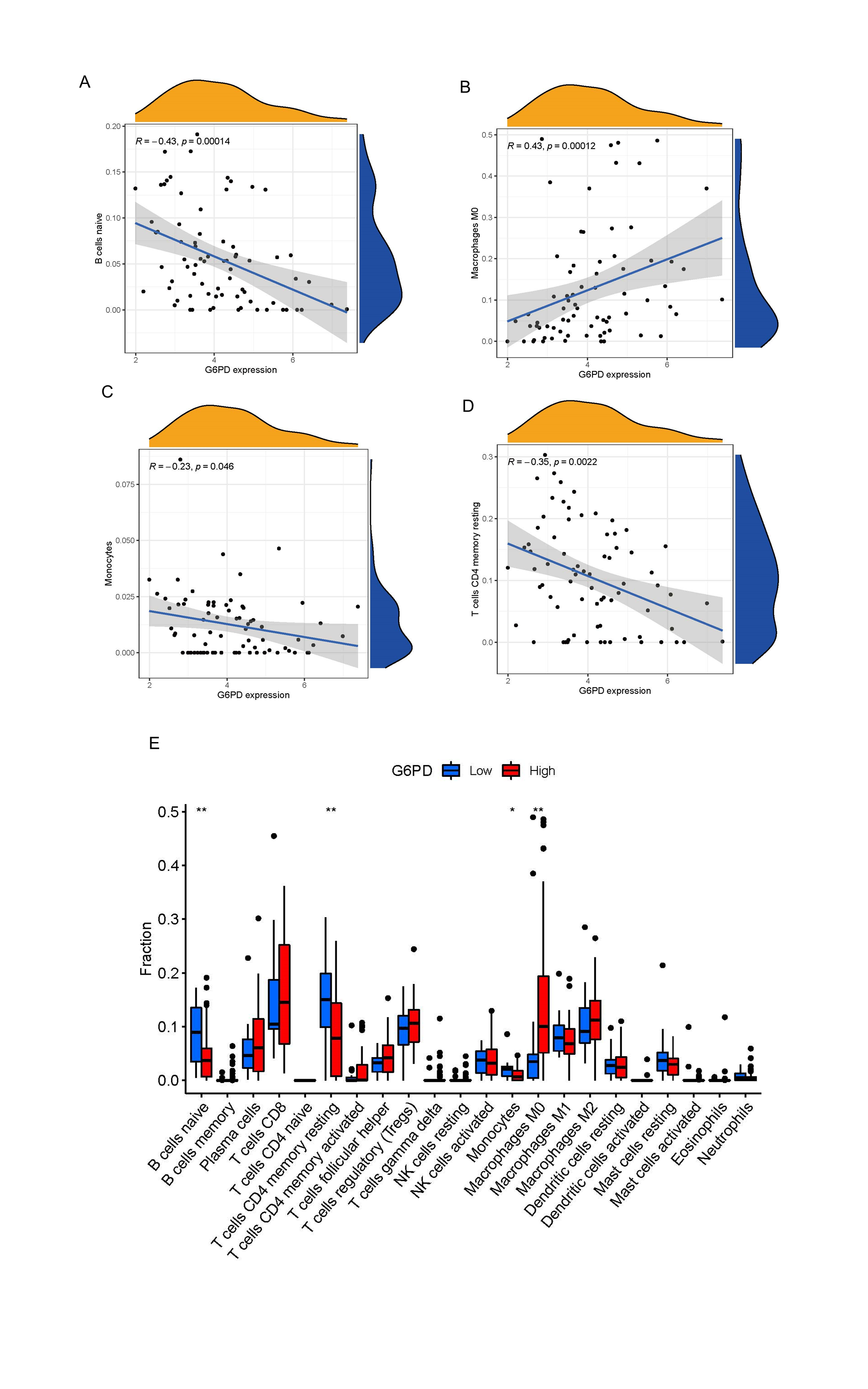
**

**Fig. S6**Immunity Analyses. (A-E) Relationship between *G6PD* expression level and immune cells. (F) Relationship between *G6PD* expression level and immune checkpoint. *P < 0.05, **P < 0.01, ***P < 0.001.

**WB-images**

FIGURE2-WB

1. WRL68-SNU398-SNU449-SKHPE1-LI7-actin


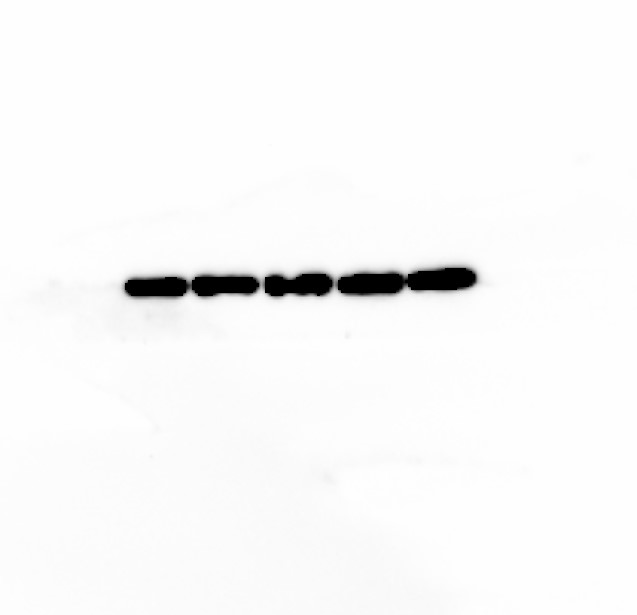


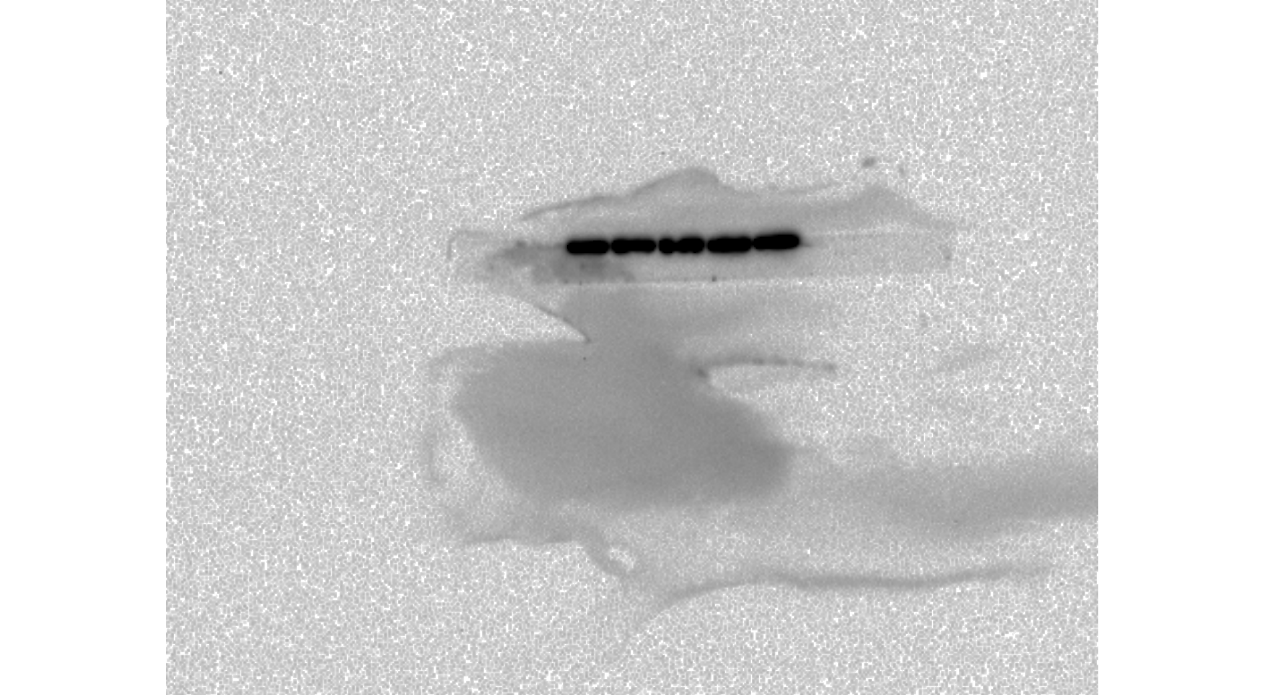


1. WRL68-SNU398-SNU449-SKHPE1-LI7-G6PD


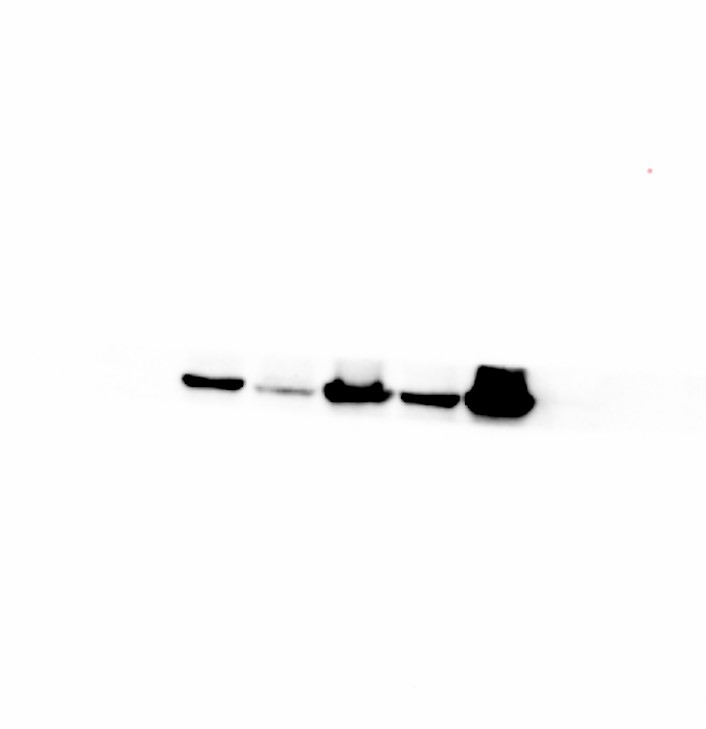


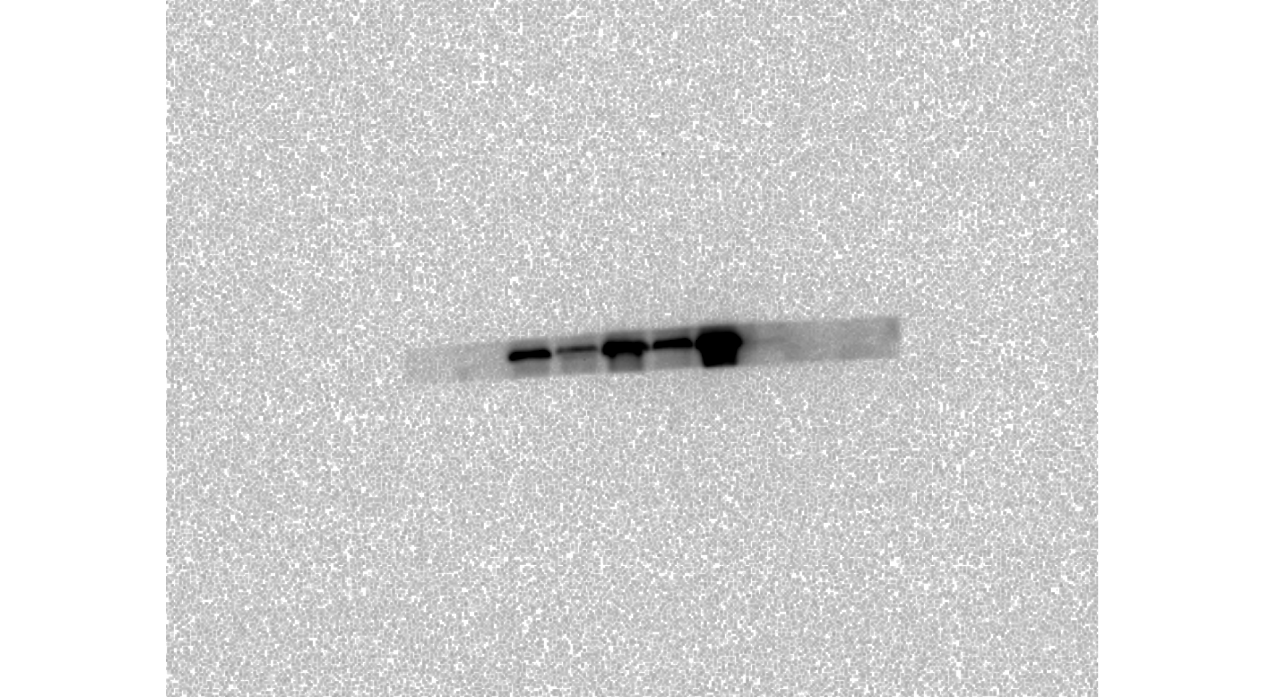


FIGURE6-WB

1. shNC-LI7, shG6PD-LI7, ACTIN


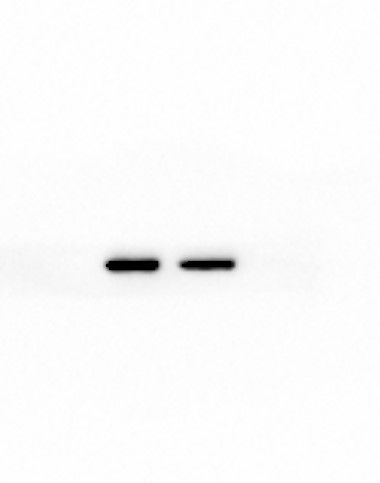


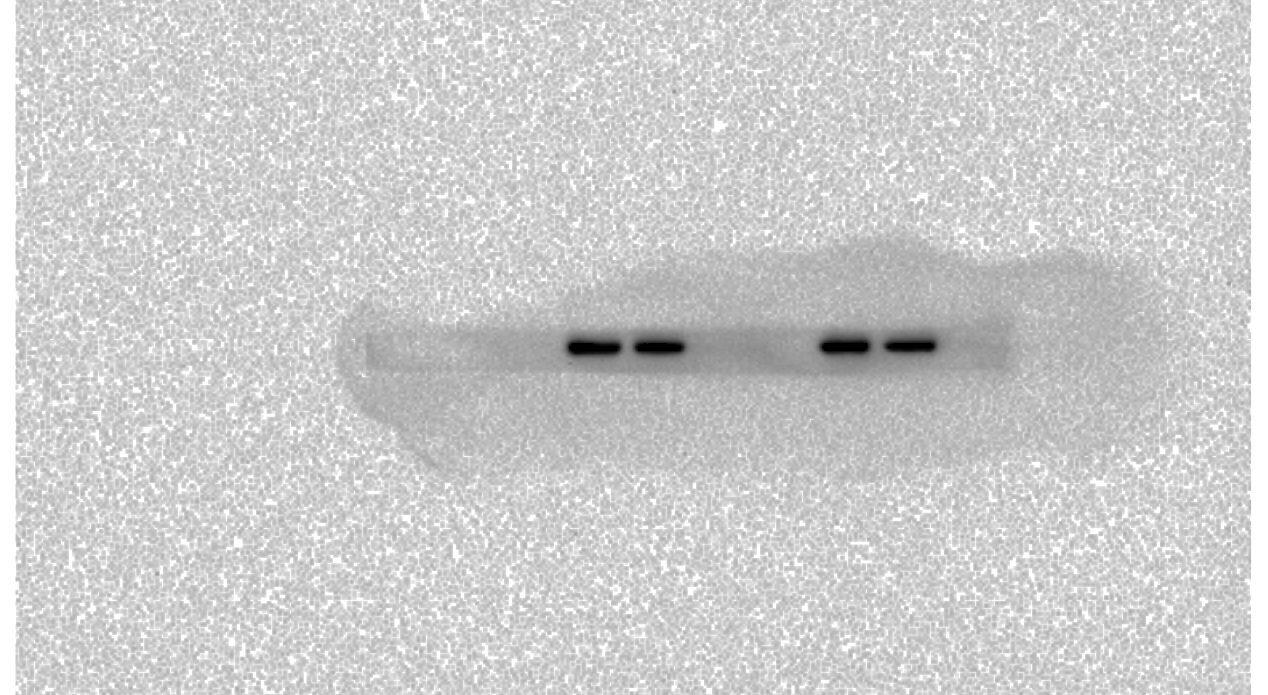


1. shNC-LI7, shG6PD-LI7, G6PD


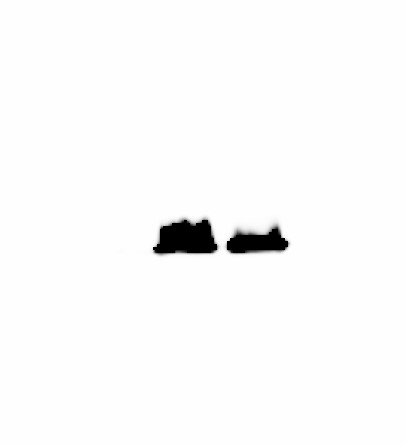


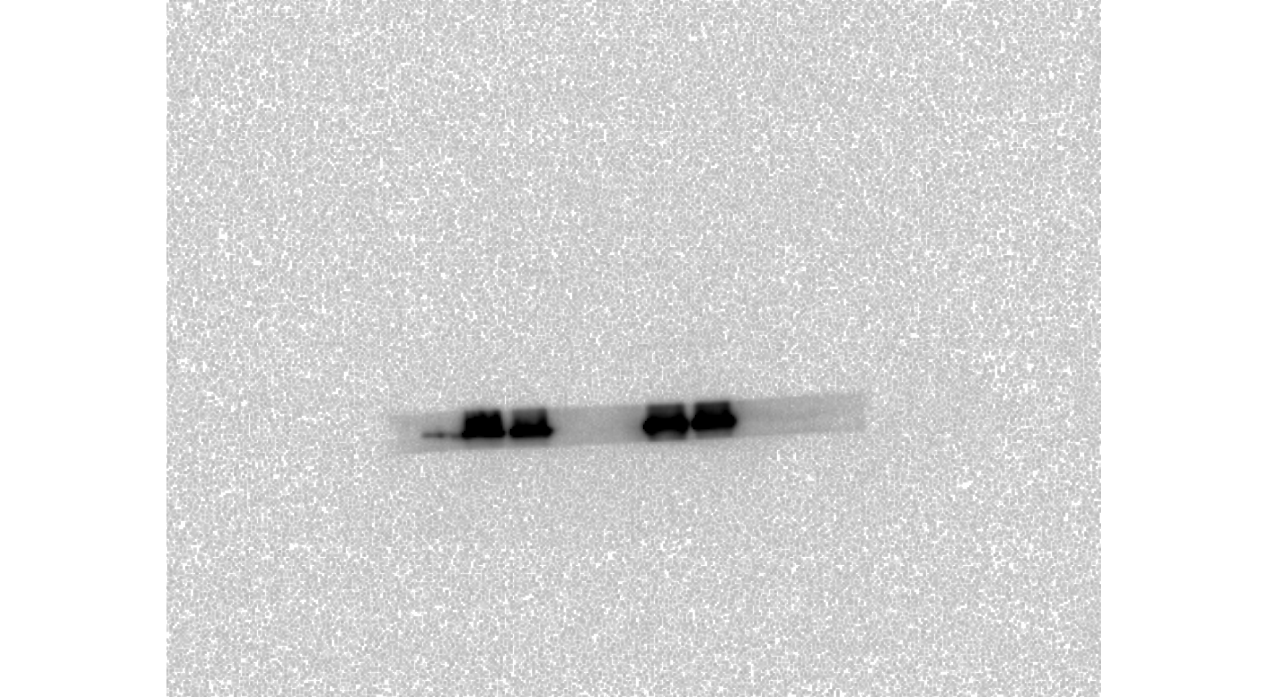


1. shNC-SNU449, shG6PD-SNU449, ACTIN


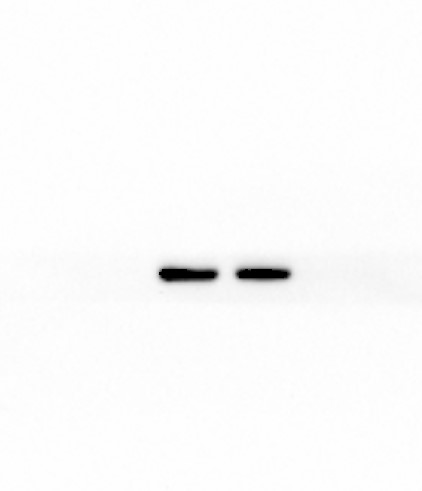


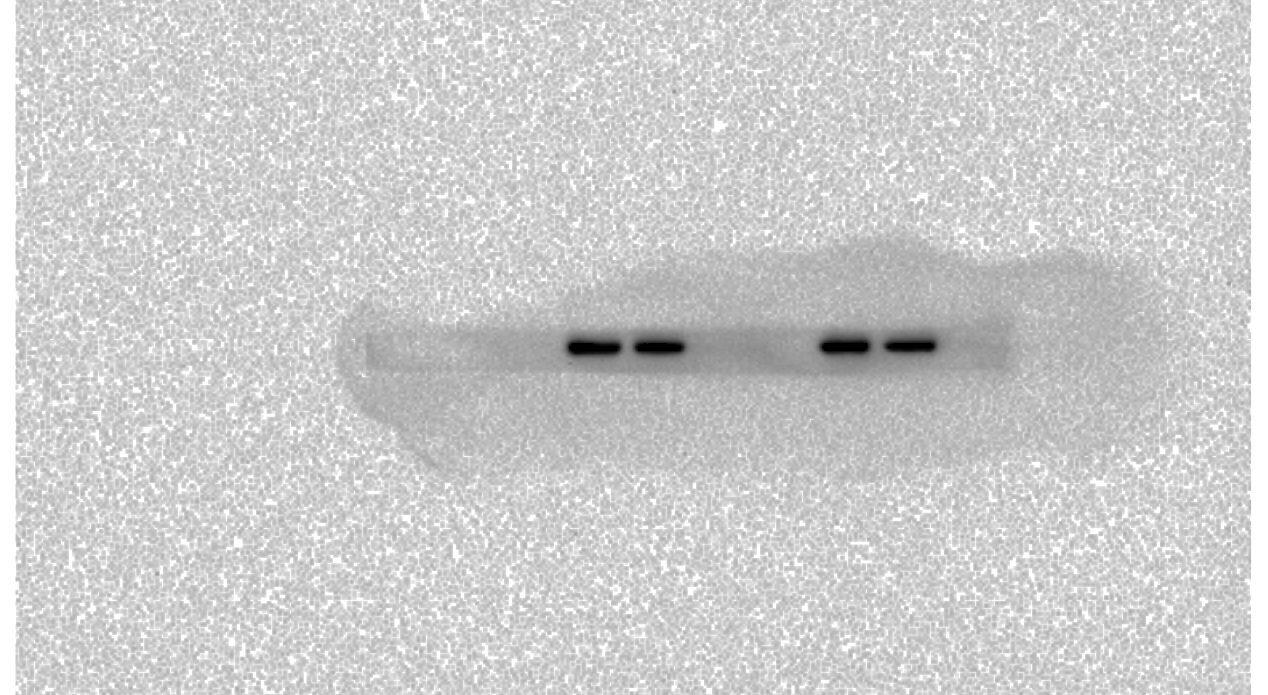


1. shNC-SNU449, shG6PD-SNU449, G6PD


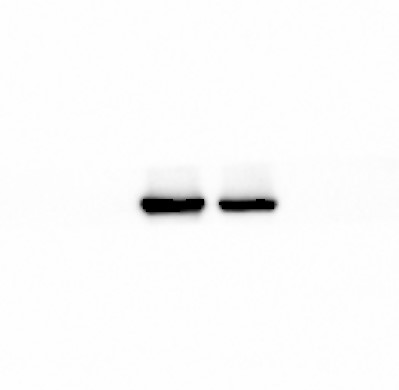


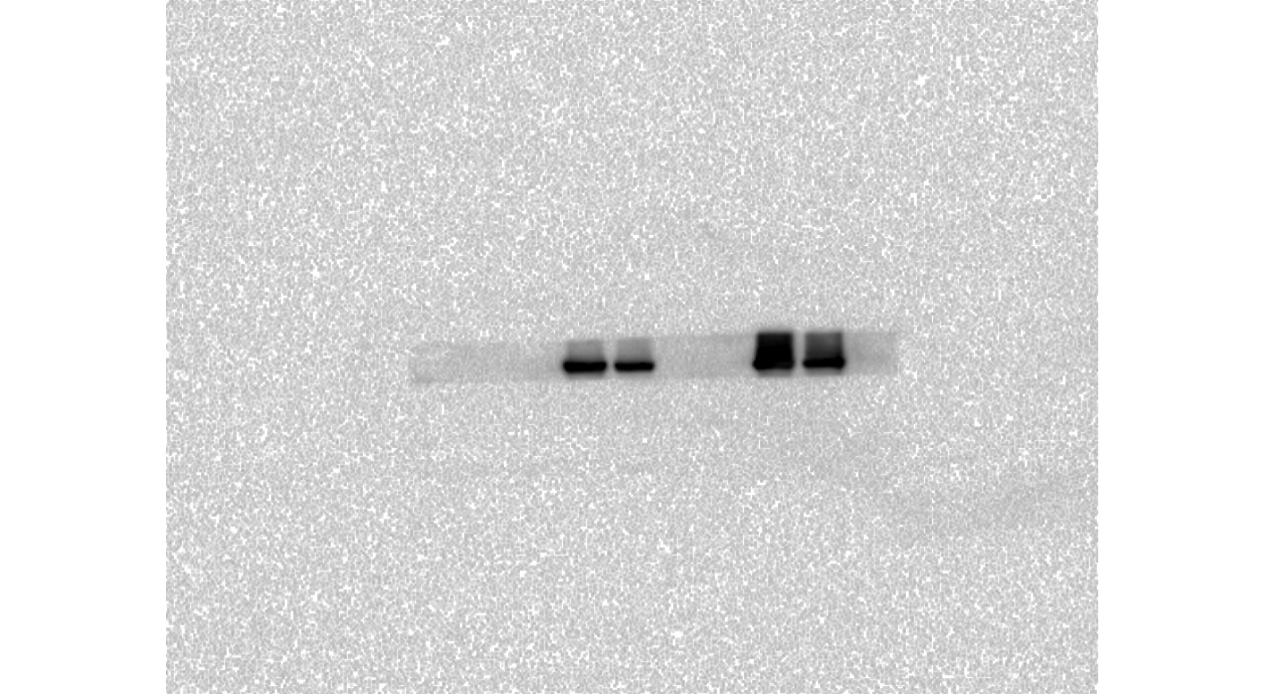

Supplement: Supplementary file 1 — Additional file 1. [file 12885_2024_11887_MOESM1_ESM.zip › Supplementary Material.docx]
