# Supplementary figures and images for: G6PD and machine learning algorithms as prognostic and diagnostic indicators of liver hepatocellular carcinoma
Source: BMC Cancer. 2024 Jan 31;24:157. doi: 10.1186/s12885-024-11887-6 (PMC10829225; doi:10.1186/s12885-024-11887-6)

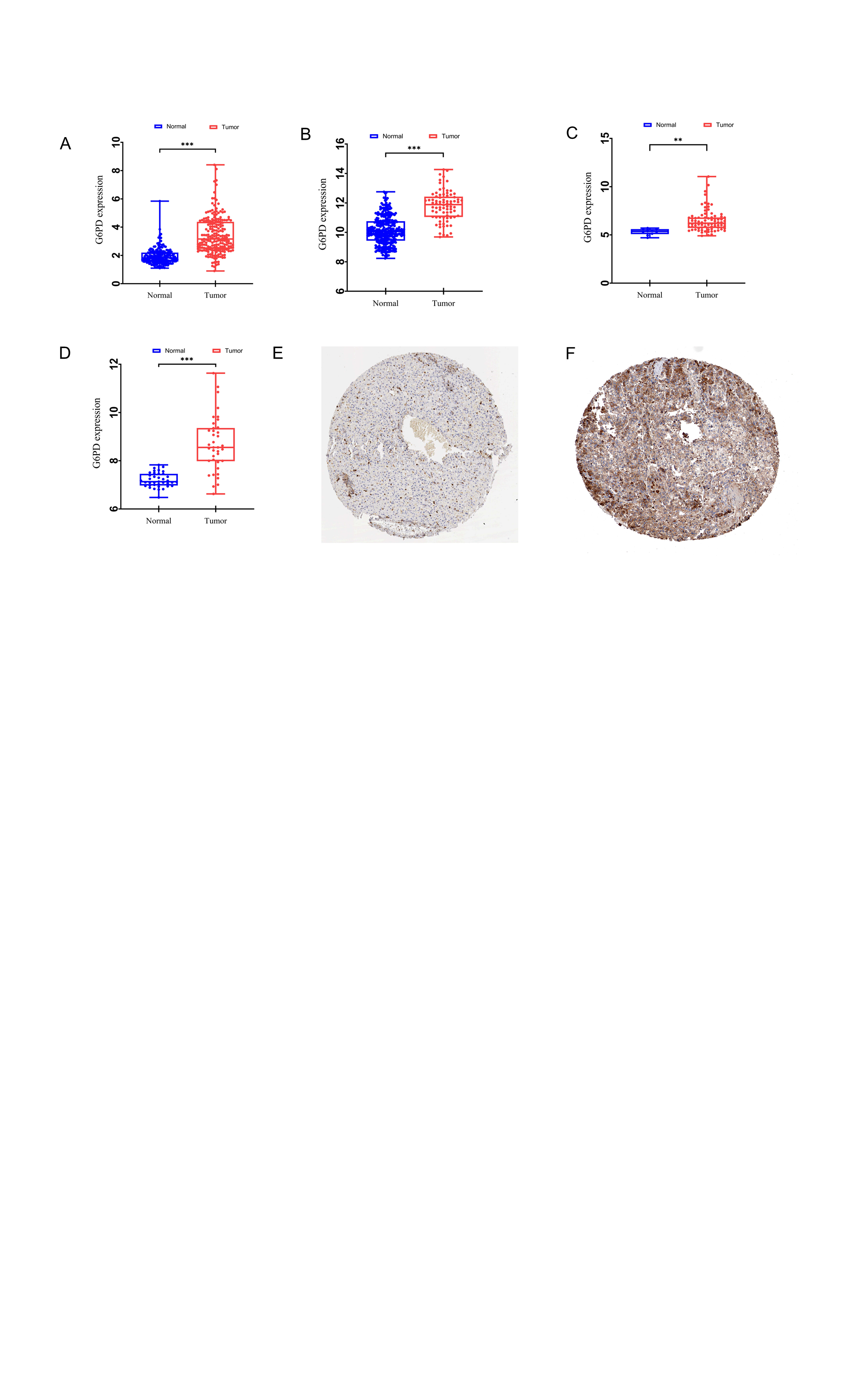

Supplement: Supplementary file 1 — Additional file 1. [file 12885_2024_11887_MOESM1_ESM.zip › FIGURE S1.TIF]

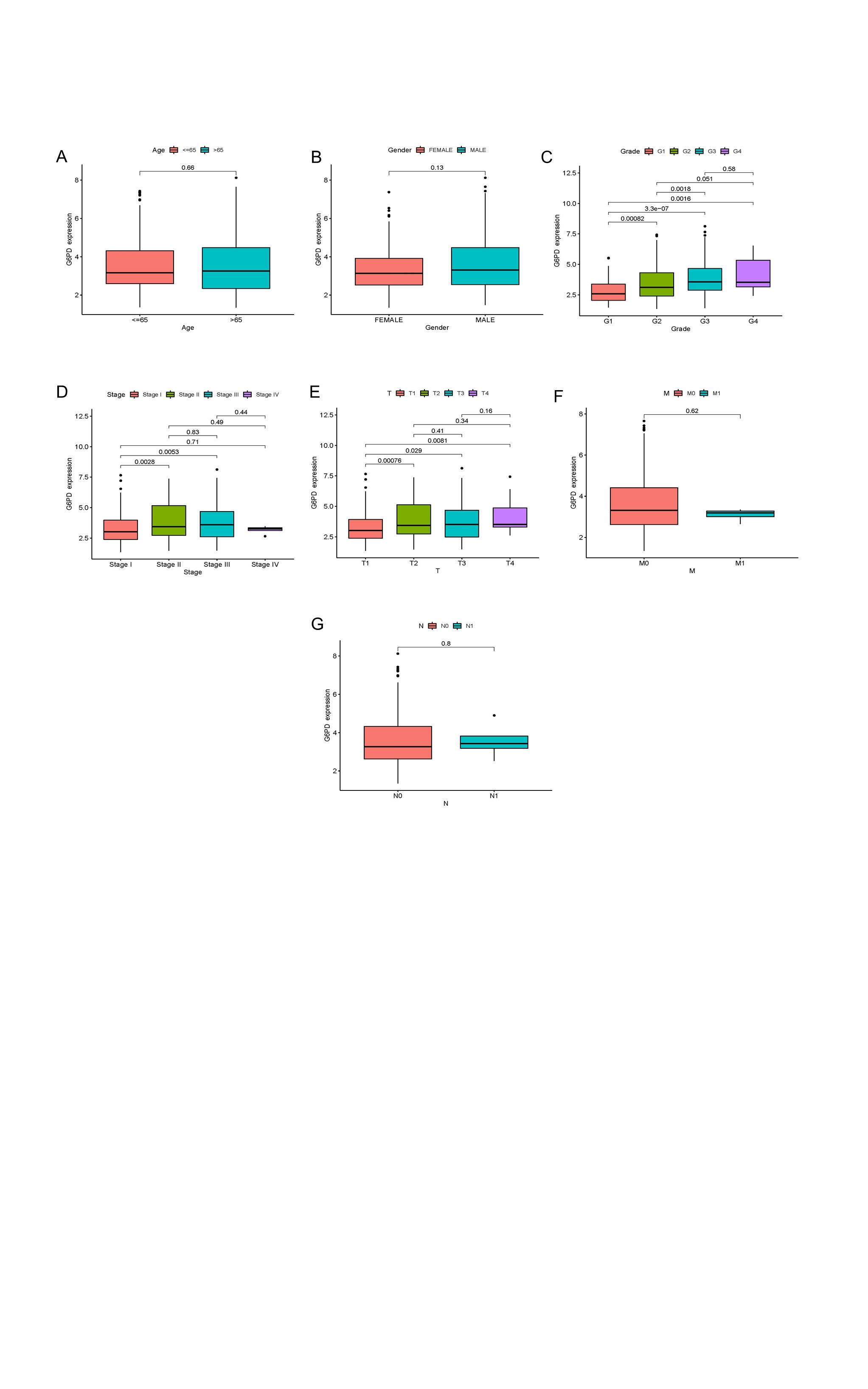

Supplement: Supplementary file 1 — Additional file 1. [file 12885_2024_11887_MOESM1_ESM.zip › FIGURE S2.TIF]

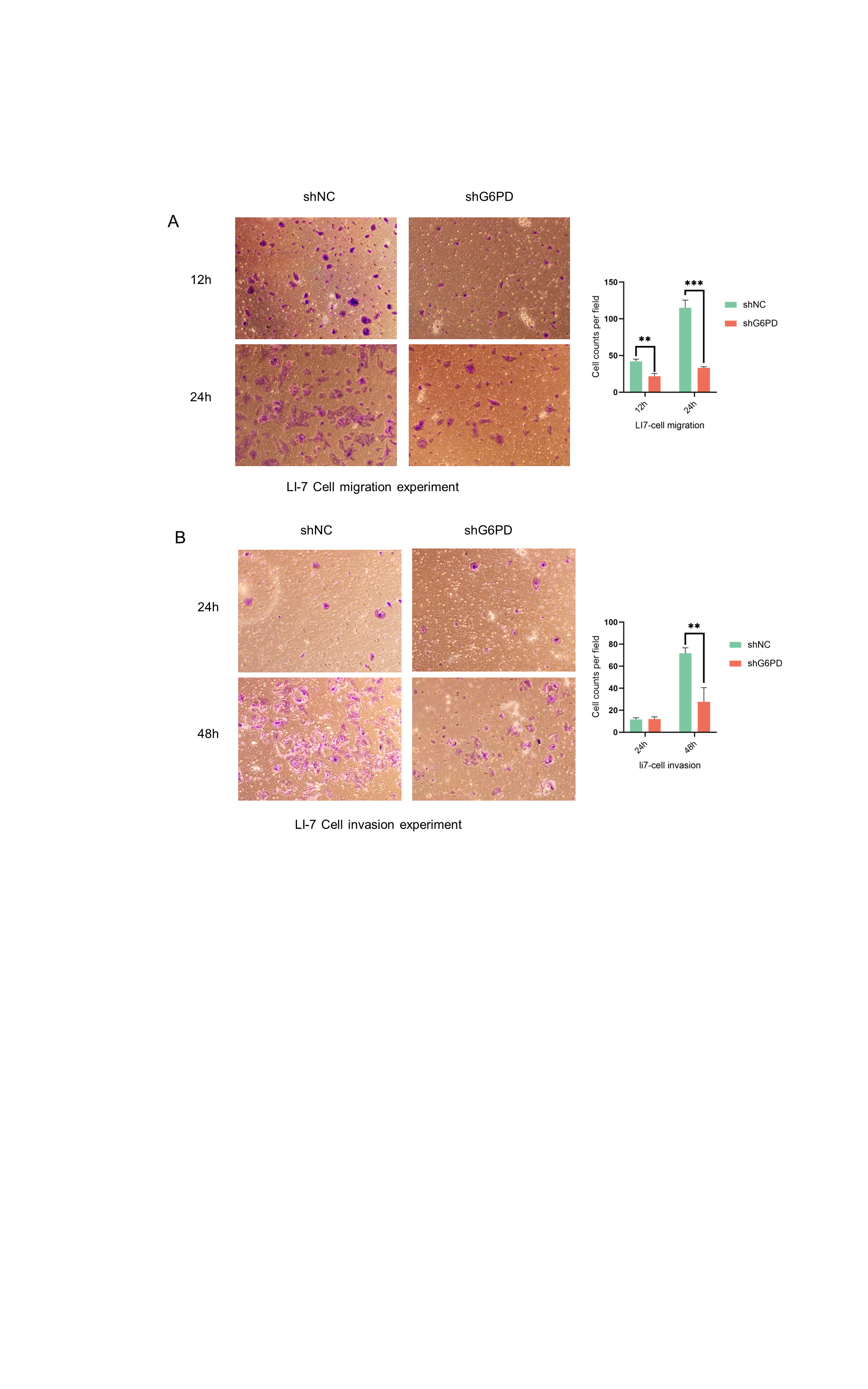

Supplement: Supplementary file 1 — Additional file 1. [file 12885_2024_11887_MOESM1_ESM.zip › FIGURE S3.TIF]

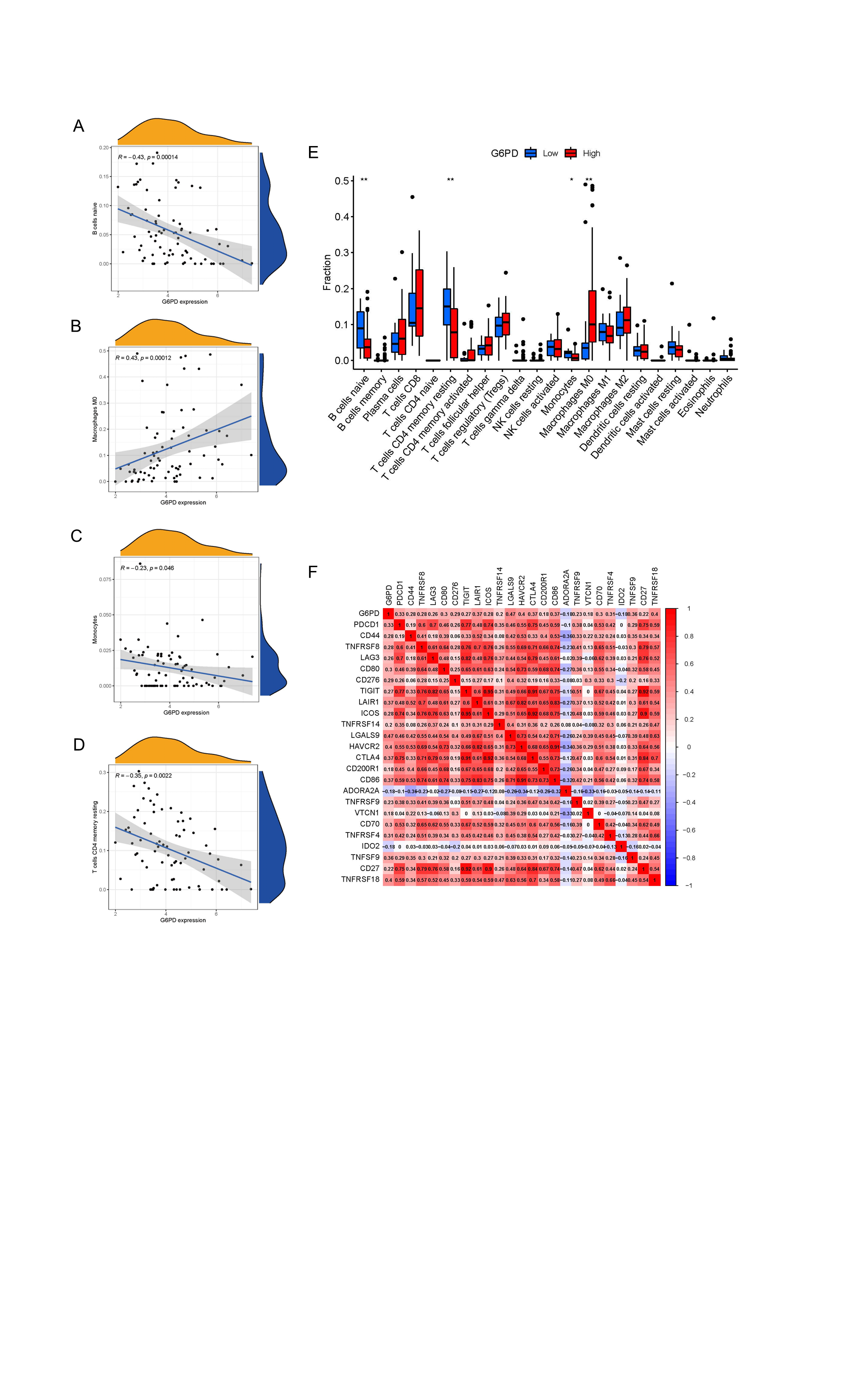

Supplement: Supplementary file 1 — Additional file 1. [file 12885_2024_11887_MOESM1_ESM.zip › FIGURE S4.TIF]
